# The dynamic immune response of the liver and spleen in leopard coral grouper (*Plectropomus leopardus*) to *Vibrio harveyi* infection based on transcriptome analysis

**DOI:** 10.3389/fimmu.2024.1457745

**Published:** 2024-10-10

**Authors:** Yang Liu, Sheng Lu, Mengqi Guo, Ziyuan Wang, Bowen Hu, Bo Zhou, Songlin Chen

**Affiliations:** ^1^ State Key Laboratory of Mariculture Biobreeding and Sustainable Goods, Yellow Sea Fisheries Research Institute, Chinese Academy of Fishery Sciences, Qingdao, Shandong, China; ^2^ Laboratory for Marine Fisheries Science and Food Production Processes, Qingdao Marine Science and Technology Center, Qingdao, Shandong, China; ^3^ Wanning Linlan Aquaculture Co., LTD, Wanning, Hainan, China

**Keywords:** *Plectropomus leopardus*, *Vibrio harveyi*, transcriptome, dynamic immune regulation, tissue-specific immune response

## Abstract

Leopard coral grouper (*Plectropomus leopardus*) is one of the most important cultured fish in the Pacific and Indian oceans. *Vibrio harveyi* is a serious pathogen causing serious skin ulceration and high mortality in *P. leopardus*. To gain more insight into the tissue-specific and dynamic immune regulation process of *P. leopardus* in response to *V. harveyi* infection, RNA sequencing (RNA-seq) was used to examine the transcriptome profiles in the spleen and liver at 0, 6, 12, 24, 48, and 72 h post-infection. The upregulated differentially expressed genes (DEGs) were predominantly involved in the immune response in the spleen and liver at the early infection stage (6–12 h), and downregulated DEGs were mainly involved in metabolic processes in the liver at the early and middle infection stage (6–48 h). Moreover, an overview of the immune response of *P. leopardus* against *V. harveyi* was exhibited including innate and adaptive immune-related pathways. Afterwards, the results of WGCNA analysis in the spleen indicated that TAP2, IRF1, SOCS1, and CFLAR were the hub genes closely involved in immune regulation in the gene co-expression network. This study provides a global picture of *V. harveyi*-induced gene expression profiles of *P. leopardus* at the transcriptome level and uncovers a set of key immune pathways and genes closely linked to *V. harveyi* infection, which will lay a foundation for further study the immune regulation of bacterial diseases in *P. leopardus*.

## Introduction

Due to high nutritional value, appealing body color, and considerable culturing profit, the leopard coral grouper (*Plectropomus leopardus*) has become a popular marine fish for aquaculture in many Asian countries (1–3). However, the shortage of aquaculture waters makes intensive, and high-density aquaculture mode commonly used in *P. leopardus* aquaculture, and what follows is the concentrated outbreak of diseases (4). At present, the skin ulceration caused by *Vibrio harveyi* is one of the most serious diseases faced by *P. leopardus*, which could destroy the skin mucus immunity, the first barrier of innate immunity system, thereby cause surface ulceration of *P. leopardus* (5). Following succeeding in the breach of mucosal epithelial barriers, invading pathogens can deepen infection processes in inflammatory foci of internal tissues in fish, even lead to death (6). Therefore, elucidating the immune defense mechanisms of *P. leopardus* against *V. harveyi* is important for developing effective strategies for treatment and prevention of skin ulceration disease.

To prevent and control the vibriosis caused by *V. harveyi*, many studies have focused on the analysis of interaction between *V. harveyi* and fish to explore the infection mechanisms and the immune defense of fish. For example, Zhang et al. (2022) reported the dynamic changes of gene expression patterns in the Chinese tongue sole kidney after *V. harveyi* infection and revealed that the Jak-STAT signaling pathway played a crucial role in the regulation during *V. harveyi* infection (6). In *Centropristis striata* challenged by *V. harveyi*, numerous mRNA–miRNA interactions were identified, which provide insight into the immune reactions that occur during the antimicrobial process (7). Wang et al. (2024) found that some metabolic pathways of *P. leopardus* showed adjustments in response to vibrio infection, further strengthened the intestinal chemical barrier (8). To date, there were few studies on immune response of *P. leopardus* against *V. harveyi* infection, which increase the difficulties of using immunological methods to prevent and control vibriosis caused by *V. harveyi* during the cultivation.

The spleen is a major secondary lymphoid organ in fish to filter pathogens carried by blood and is the main place to resist many micro-organisms (9). It contains diverse resident immune cell populations (e.g., lymphocytes, macrophages, and granulocytes) that secrete cytokines to propagate the inflammatory response and pathogen clearance during inflammation (10). Additionally, the spleen plays an important role in antigen presentation and adaptive immune response, which is mainly involved in the development of B cells, antigen processing, and MHC class II molecule expression (11). Fish liver is classically perceived as a non-immunological organ that functions primarily in metabolic processes, detoxification, and nutrient storage. In fact, the liver is generally responsible for generating acute-phase proteins (e.g., C-reactive protein, complement proteins, serum amyloid A), cytokines, and pattern-recognition receptors (PRRs), which were identified as an essential immune organ in fish (12). The liver’s dual roles in both immune and metabolism function make it an interesting candidate to study the dialogue between fish immunity and metabolism upon pathogen challenges. The intimate interactions between these two seemingly unrelated systems remain enigmatic for long time. Sharing or competition of energy was thought to be one of possible ways to govern immune-metabolic interactions (13). Rauw (2012) reported that sufficient energy reserves are required to maintain the immune function and activate defense responses (14). Hence, it is necessary to study the interactions between the immune and metabolic system through the fish spleen and liver.

Characterizing similar or differential gene expression patterns among immune organs during the pathogenic infection may offer a more integrated approach in immunological studies. For example, Floreste et al. (2023) found that the proinflammatory cytokine expression was more pronounced in the liver that in the spleen at earliest time points in the lipopolysaccharide (LPS)–challenged *Rhinella diptycha* (10). Fonseca et al. (2021) showed that tumor necrosis factor (TNF) production was driven by a spleen–liver axis in a rat model of systemic inflammation induced by bacterial LPS and implicated LTB4 as a spleen-derived endocrine signal that promoted the hepatic production of TNF during systemic inflammation, providing a framework to understand how systemic inflammation can be regulated at the level of interorgan communication (15). It can be inferred that critical pathogens for fish may also alter immune gene expression in a time- and organ-related manner. Therefore, a time course of organ-integrative characterization of the immune response in fish is urgently required.

Transcriptome profiling is a powerful tool to show gene expression patterns in immune organ during the bacterial infection and has provided new insights into immune responses against bacterial infections in some target tissues of various aquaculture animals (16–19). However, there has been limited information to use transcriptome analysis to study the dynamic immune process of fish in time and space so far. To fully investigate the dynamic and organ integrative of the immune response in *P. leopardus* against *V. harveyi*, it is necessary to conduct the transcriptome analysis based on spleen and liver at different time points post-infection. In this study, the transcriptomic profiles were obtained by RNA sequencing (RNA-seq) technology from the spleen and liver tissues of *P. leopardus* at 0, 6, 12, 24, 48, and 72 h post-infection with *V. harveyi*. The results can provide new insights into the immune regulation of *P. leopardus* against *V. harveyi* and provide basic data for vibrio disease prevention in the *P. leopardus* intensive culture processes.

## Materials and methods

### Bacteria challenge and sample collection

**Figure 1:**
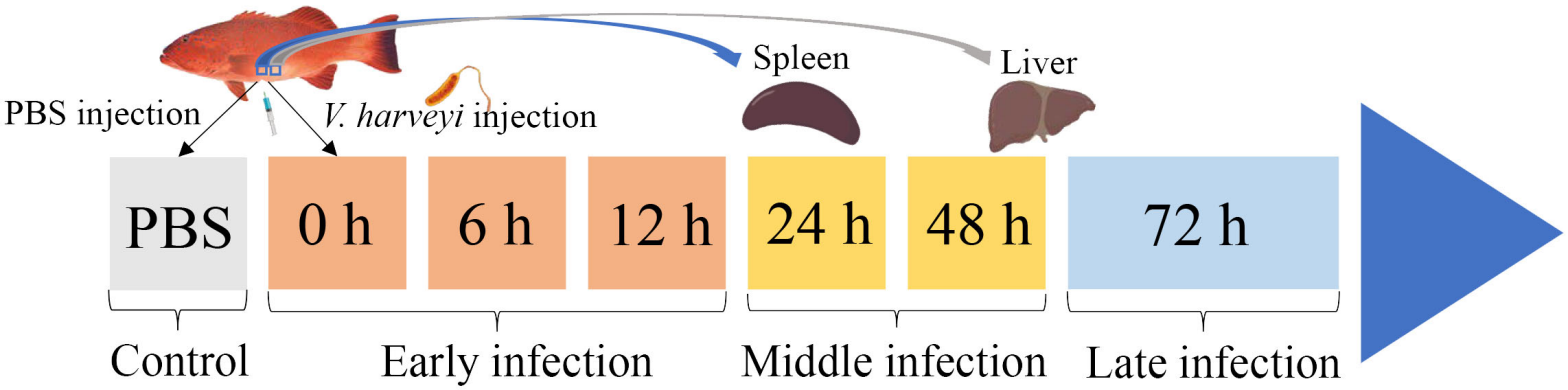
Schematic illustration of the sampling for *V. harveyi* infection experiment in *P. leopardus*. After the intraperitoneal injection of *V. harveyi*, the spleen and liver were sampled at 0, 6, 12, 24, 48, and 72 h. The control group were sampled at the beginning after the intraperitoneal injection of PBS. The six sampling points with *V. harveyi* infection were further divided into early infection stage (0, 6, and 12 h), middle infection stage (24 and 48 h), and late infection stage (72 h).

Two hundred healthy juvenile *P. leopardus* with body weights of 15.0 ± 3.3 g were obtained from Mingbo Aquatic Company (Laizhou, China). The fish were maintained in tanks containing aerated sand-filtered seawater at 23 ± 0.5°C for 1 week prior to processing. *V. harveyi* for immune challenge experiment was isolated from diseased fish and kept in our laboratory (5). In brief, the bacteria were incubated to mid-logarithmic stage at 28°C in tryptic soy broth medium, then collected by centrifugation and re-suspended in phosphate-buffered saline (PBS, pH 7.2) to a final concentration of 1 × 10^5^ CFU/ml (20). Fifty fish intraperitoneally injected with live *V. harveyi* suspension at 0.1 ml/100 g fish weight were set as the infected group. The remaining 50 fishes injected with PBS at 0.1 ml/100 g fish weight were set as the control group. After anesthesia in 5 mg/L MS-222 (tricaine methane sulfonate), three individual fish from the infected group were sampled at 6 time points (0, 6, 12, 24, 48, and 72 h after *V. harveyi* injection), of which the 0, 6, and 12 h were regarded as the early infection stage, 24 and 48 h were regarded as the middle infection stage, and 72 h was regarded as the late infection stage based on the regularity of mortality in the *P. leopardus* after *V. harveyi* infection in our previous study (**Figure 1**) (21). The spleen samples were named as S0h, S6h, S12h, S24h, S48h, and S72h, respectively. The liver samples were named as L0h, L6h, L12h, L24h, L48h, and L72h, respectively. All samples were transferred into liquid nitrogen and stored at −80°C.

### RNA extraction and sequencing

Total RNA was extracted from liver and spleen tissues using Trizol Reagent (Invitrogen, Carlsbad, California, USA) according to the manufacturer’s protocol. The quality, purity, concentration, and integrity of RNAs were detected using the agarose gel electrophoresis, Nanodrop ND-1000, Qubit 2.0, and Agilent 2100 RNA 6000 Nano kit (Agilent, USA). Then, the cDNA libraries were constructed using 3 μg qualified total RNA (RIN > 7.0) via the conventional protocol, and subsequent sequencing was conducted with an Illumina NovaSeq by Gene Denovo Biotechnology Co., China.

### Transcriptomic analysis

The raw reads were filtered by fastp (version 0.18.0), and then the clean data were mapped to *P. leopardus* reference genome via HISAT (v2.2.4) (22). Then, transcript assemblies were conducted with StringTie (v1.3.1) and Cufflinks (v2.2.1) in a reference-based approach. To investigate the differential responses of *P. leopardus* to *V. harveyi* infection, the number and biological functions of differentially expressed genes (DEGs) in different groups were analyzed. FPKM (fragment per kilobase of transcript per million mapped reads) value was calculated to quantify the gene expression levels with RSEM software. DEGs were identified using DESeq2 package with the default parameter. The |log_2_(fold change)| ≥ 1 and *Q*-value (adjusted *P*-value) ≤ 0.05 were used as the filtering thresholds. Further, the biological function of DEGs were annotated using Gene Ontology (GO) (Blast2GO) and Kyoto Encyclopedia of Genes and Genomes (KEGG) (KOBAS) database. The enrichment analyses of GO and KEGG were performed using Goatools and Python softwares, respectively. The hyper-geometric distribution corrected *Q*-value < 0.05 was taken as the criterion of significant enrichment of the GO term and KEGG pathway.

### Weighted gene co-expression network analysis

A weighted gene co-expression network analysis (WGCNA) was performed using the R package BioNERO (v1.6.1), following the published method (23). After filtering genes (FPKM < 1.5 in half or more of the samples), gene expression values were imported into BioNERO to (i) remove missing data, (ii) remove genes with low expression across samples, (iii) remove outliers, and (iv) remove confounders that could introduce false-positive correlations. To make the Gene Co-Expression Networks (GCNs) satisfy the scale-free topology, of which the scale-free topology fit index (*R* ^2^) reaches 0.8 and the mean connectivity tends to 0, we identified the best β power with the function of SFT_fit. Then, the GCN was inferred using the exp2gcn function with β power. Eventually, co-expression modules and hub genes were identified, and GO and KEGG analyses were performed on these responsive modules to elucidate their biological functions. Related networks were visualized using Cytoscape v3.10.2.

### Quantitative real-time polymerase chain reaction analysis

The candidate genes screened by transcriptomic analysis were subjected to quantitative real-time polymerase chain reaction (qRT-PCR) for validation. qRT-PCR was performed using a 7500 fast RT-PCR system (Applied Biosystems, Foster City, CA, USA) with SYBR green kit (Takara, Kyoto, Japan). In brief, β-actin served as the internal reference gene, and the relative gene expression values were calculated by the 2^–ΔΔCt^ method. SPSS 18.0 software (IBM) was used for sample comparisons via Duncan’s test, and a significant difference was observed at *P* ≤ 0.05. The primer sequences for these genes are listed in **Supplementary Table S1**.

## Results

### Transcriptome sequencing data

To understand the dynamic immune mechanisms of *P. leopardus* from the spleen and liver in response to *V. harveyi* infection, we performed transcriptome sequencing of a total of 42 samples. The RNA-seq data were deposited in the National Center for Biotechnology Information under accession number: PRJNA1126611. By comparing the unigenes from the different time points after *V. harveyi* infection with that from the control PBS based on the criteria for DEGs, large number of DEGs were concentrated at 6–24 h in both spleen and liver, especially at the early infection stage 6–12 h. At 0 and 72 h, the number of downregulated DEGs was more than that of upregulated DEGs in both spleen and liver (**Table 1**). Venn diagrams showed that the proportions of liver–spleen common DEGs at 6, 12, and 24 h were more than 20%, and the proportions of spleen-specific DEGs at 48 and 72 h were up to 48.3% and 50.2%, respectively (**Supplementary Figure S1**).

**Table 1:** The number of DEGs in the spleen and liver of *P. leopardus* at different time points after *V. harveyi* infection.

Time	Spleen			Liver		
	Up	Down	Total	Up	Down	Total
0 h	53	137	190	85	206	291
6 h	1881	2696	4577	2364	2089	4453
12 h	1838	2436	4274	2216	2406	4622
24 h	1359	1329	2688	1532	1430	2962
48 h	426	514	940	219	603	822
72 h	299	445	744	180	452	632

### Function analysis of all DEGs

To investigate the dynamic change of the immune functions at different time points after infection, GO and KEGG enrichments were performed based on the DEGs from the spleen and liver, respectively. Ten most enriched GO terms were selected in the spleen and liver at 0–72 h after infection, respectively (**Supplementary Figure S2**, **Supplementary Table S2**). Major GO terms were distributed in the biological processes in both spleen and liver. In the spleen, immune response-associated GO terms were significantly detected at 6 h, and cell cycle process associated GO terms were found at 48 to 72 h (**Supplementary Figure S2A**). In the liver, metabolic process associated GO terms were detected from 0 h to 24 h, and response to organic cyclic compound was found at 48 h (**Supplementary Figure S2B**).

**Figure 2:**
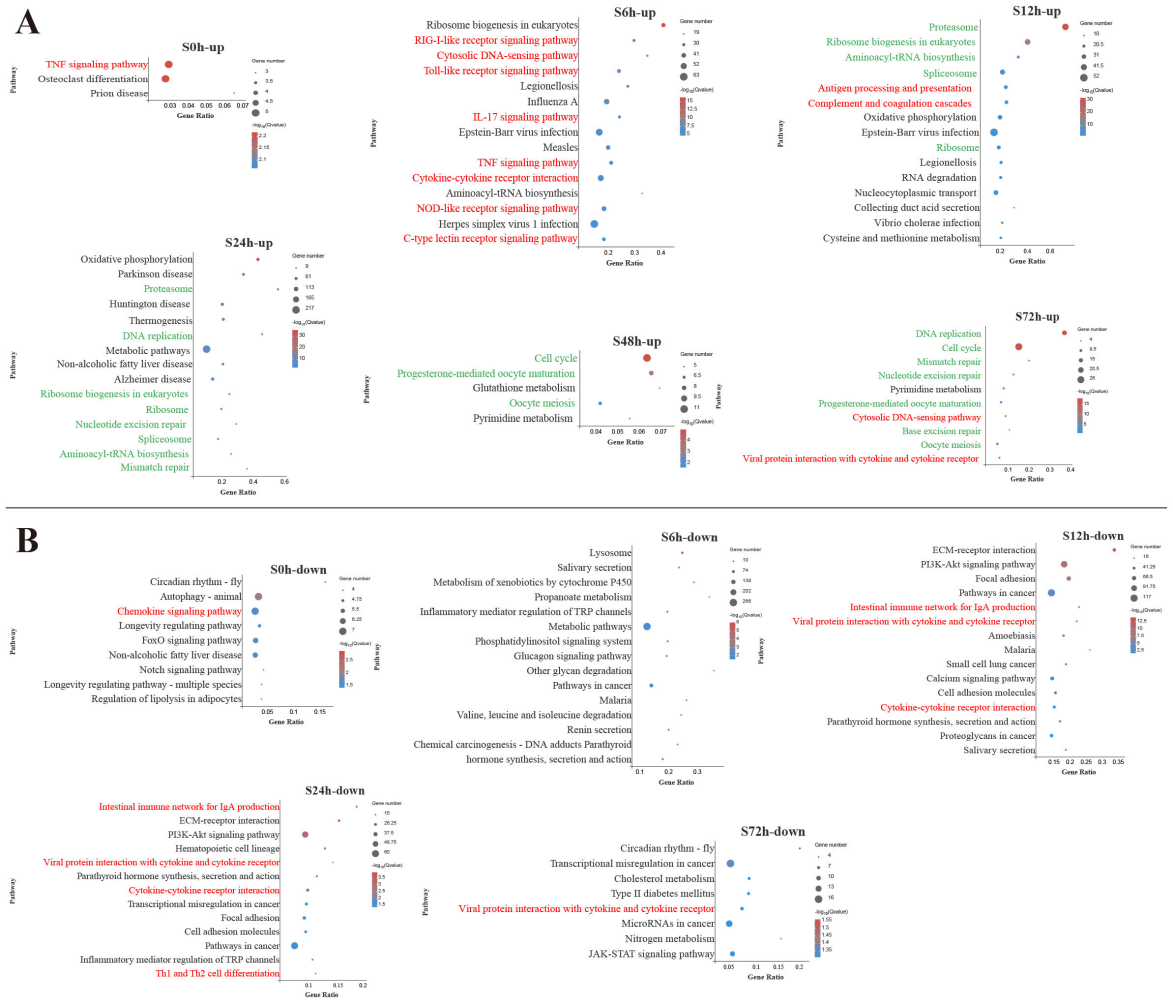
Kyoto Encyclopedia of Genes and Genomes (KEGG) enrichment analysis of upregulated **(A)** and downregulated **(B)** differentially expressed genes (DEGs) in the spleen. Red font and green font represent immune- and cellular and genetic information process-related pathways, respectively. The vertical coordinate represents the enriched KEGG pathways, and the horizontal coordinate represents the Rich factor. The size of the dots represents the number of DEGs in KEGG pathways, and different colors of dots represent the different log_2_ (*Q*-values).

**Figure 3:**
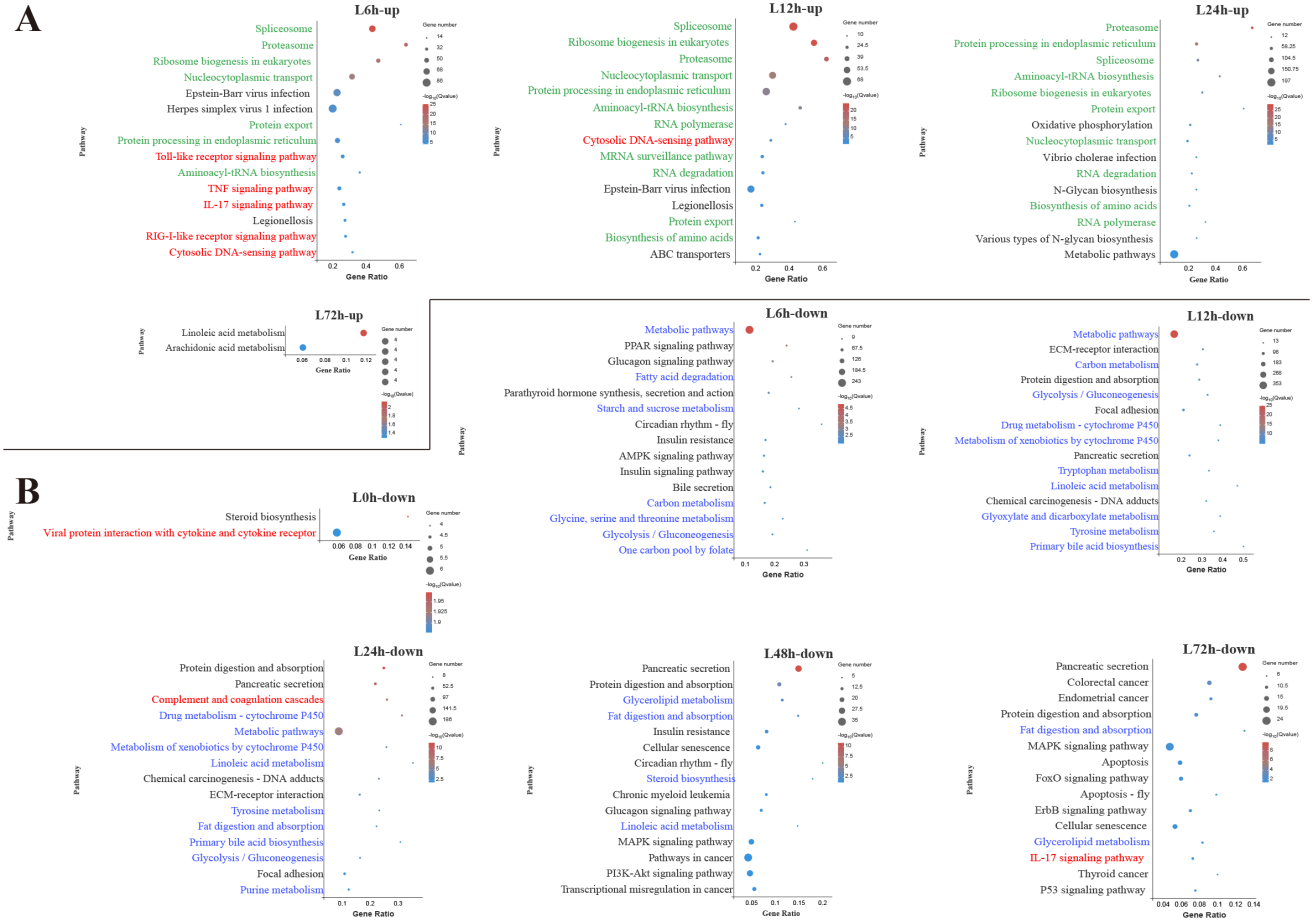
Kyoto Encyclopedia of Genes and Genomes (KEGG) enrichment analysis of upregulated **(A)** and downregulated **(B)** differentially expressed genes (DEGs) in the liver. Red font, blue font, and green font represent immune-, metabolic-, and cellular and genetic information process-related pathways, respectively. The vertical coordinate represents the enriched KEGG pathways, and the horizontal coordinate represents the Rich factor. The size of the dots represents the number of DEGs in KEGG pathways, and different colors of dots represent the different log_2_ (*Q*-values).

To identify the biological pathways involved in *V. harveyi* infection, the DEGs enrichment analysis using KEGG database were conducted (**Supplementary Table S3**). **Figures 2** and **3** display the significantly enriched KEGG pathways in the spleen and liver at different infection time, respectively. In the spleen, most upregulated DEGs were enriched in the pathways related to the immune response at the early infection stage and the cellular process at the middle infection stage and the late infection stage (**Figure 2A**). These immune response pathways mainly related to the innate immune system, such as PRR-related pathways (RIG-I-like receptor signaling pathway, Toll-like receptor (TLR) signaling pathway, NOD-like receptor signaling pathway, and C-type lectin receptor signaling pathway) and inflammatory response-related pathways [interleukin-17 (IL-17) signaling pathway, TNF signaling pathway, cytokine–cytokine receptor interaction] at 6 h and complement and coagulation cascades pathway at 12 h. In addition, the adaptive immune pathway (antigen processing and presentation) was significantly enriched at 12 h. Afterward, the upregulated DEGs in the spleen were significantly enriched in the pathways related to cellular processes and DNA repair. Among the pathways enriched by downregulated DEGs in the spleen, two adaptive immune pathways (intestinal immune network for IgA production and Th1 and Th2 cell differentiation) were significantly affected at 12–24 h (**Figure 2B**).

In the liver, among the top 15 pathways enriched by upregulated DEGs at 6 h, 5 pathways were associated with innate immune response, including TLR signaling pathway, TNF signaling pathway, IL-17 signaling pathway, RIG-I-like receptor signaling pathway and cytosolic DNA-sensing pathway (**Figure 3A**). During the early and middle infection stage (6–24 h), lots of upregulated DEGs in the liver were enriched in the pathways related to the genetic information process. When considering the significant pathways of downregulated DEGs enrichment, metabolisms were the pathways with the majority of DEGs mapped at 6–48 h. Among the metabolism-related pathways, lipid metabolism (fatty acid degradation, linoleic acid metabolism, primary bile acid biosynthesis, glycerolipid metabolism, steroid biosynthesis) was mostly affected, followed by amino acid metabolism (glycine, serine and threonine metabolism, tryptophan metabolism, tyrosine metabolism, purine metabolism) and carbohydrate metabolism (starch and sucrose metabolism, glycolysis/gluconeogenesis, glyoxylate and dicarboxylate metabolism) (**Figure 3B**).

### Immune-related pathways of *P. leopardus* against *V. harveyi* infection

**Figure 4:**
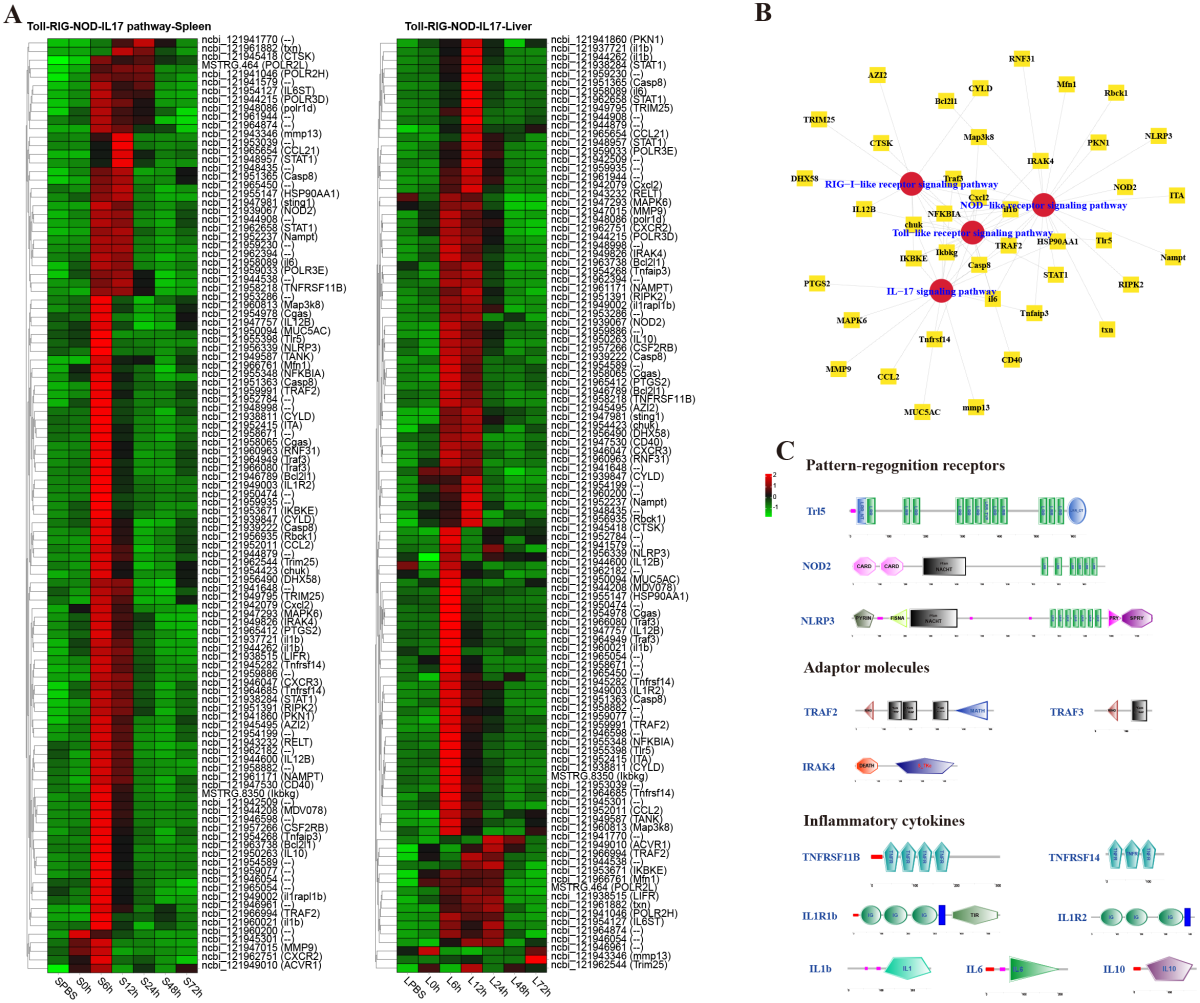
Analysis of differentially expressed genes (DEGs) in classical innate immune receptor pathways (RIG-I-like receptor signaling pathway, Toll-like receptor signaling pathway, IL-17 signaling pathway, and NOD-like receptor signaling pathway). **(A)** Heat map of DEGs in the four innate immune receptor pathways in spleen and liver. **(B)** The co-exist network of DEGs in the four innate immune receptor pathways. **(C)** Structural features of key DEGs in the innate immune receptor pathways.

To analyze the dynamic immune response of *P. leopardus* against bacterial infection, immune-related pathways were screened in the spleen and liver at different time points after infection of *V. harveyi*. The results showed that there were lots of DEGs in classical four innate immune PRR pathways (RIG-I-like receptor signaling pathway, TLR signaling pathway, IL-17 signaling pathway, and NOD-like receptor signaling pathway) found in both spleen and liver after infection (**Figure 4**). In the spleen and liver, most DEGs in the four innate immune receptor pathways were significantly upregulated at 6–12 h, especially at 6 h (**Figure 4A**). Many DEGs co-existed in different innate immune receptor pathways, for example, Ikbkg, Casp8, Cxcl2, IKBKE, chuk, NFKBIA, and Traf3 were all existed and upregulated expressed in the four innate immune receptor pathways (**Figure 4B**). The structure predictions of PRRs showed that the Tlr5 contained a signal peptide, a leucine-rich repeat (LRR) N-terminal domain, 12 LRR domains, and an LRR C-terminal domain. The NOD2 and NLRP3 all contained an NAIP, CIIA, HET-E, and TP1 (NACHT) nucleotide-binding domain and several LRR domains, while the NOD2 also had two C-terminal caspase recruitment domains, and the NLRP3 also had a fish-specific NACHT associated domain (**Figure 4C**). The structure predictions of adaptor molecules showed that TRAF2 and TRAF3 had a ring finger (RING) domain and one or two TRAF-type zinc finger (zf-TRAF) domain, and the IRAK4 contained a Serine/Threonine protein kinases, catalytic (S_TKc) domain and a DEATH domain. The structure predictions of inflammatory cytokines showed that the TNFRSF11B and TNFRSF14 contained three or four TNFR family domains. The IL1R1b and IL1R2 consisted of three IG-like domains and a transmembrane region; in addition, il1r1b contained a Toll-interleukin 1-resistance (TIR) domain. The IL-1b, IL-6 and IL-10 had a typical IL-1, IL-6, and IL-10 family domain, respectively (**Figure 4C**).

**Figure 5:**
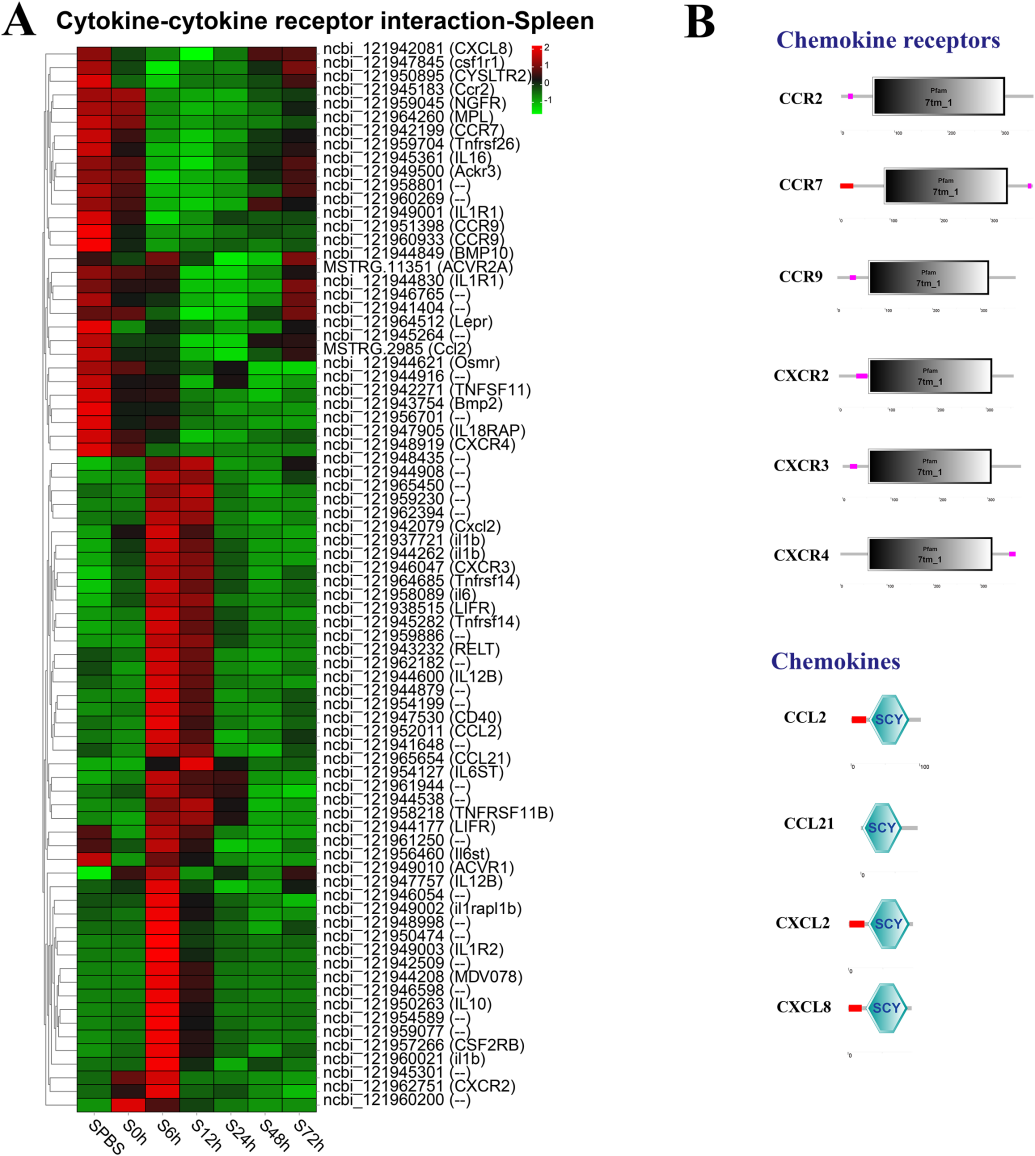
Analysis of differentially expressed genes (DEGs) in cytokine–cytokine receptor interaction pathway. **(A)** Heat map of DEGs in cytokine–cytokine receptor interaction pathway in the spleen. **(B)** Structural features of chemokine receptors and chemokines.

In the spleen, the better part of DEGs in the cytokine–cytokine receptor interaction pathway were significantly upregulated at 6–12 h, such as the CXC chemokine receptors (CXCR2 and CXCR3), and the chemokines (CCL2, CCL21, and Cxcl2), whereas the CC chemokine receptors (CCR2, CCR7, and CCR9) were notably downregulated at 6–48 h (**Figure 5A**). The structure predictions showed that all chemokine receptors had a seven-transmembrane (7tm_1) domain and all chemokines contained an intercrine alpha family (small cytokine) (SCY) domain (**Figure 5B**).

**Figure 6:**
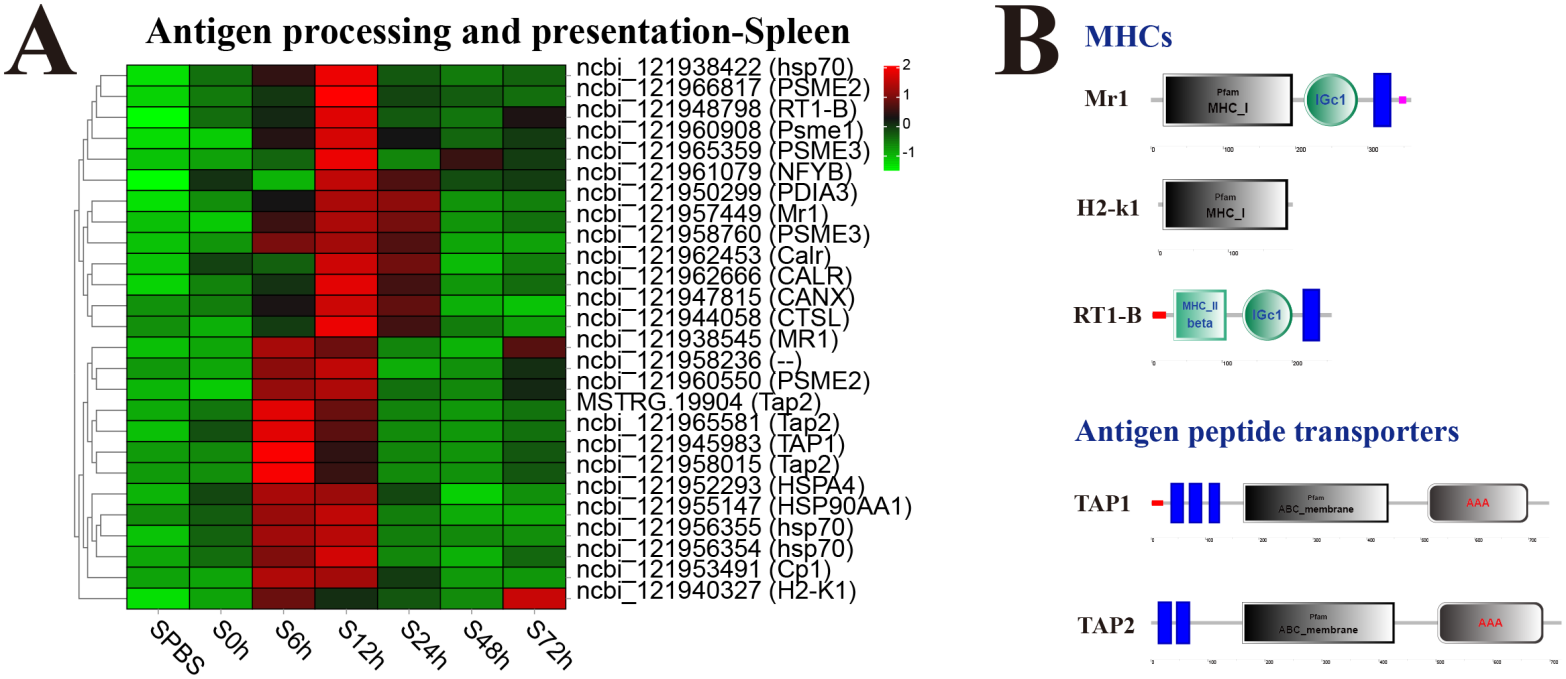
Analysis of differentially expressed genes (DEGs) in antigen processing and presentation pathway. **(A)** Heat map of DEGs in antigen processing and presentation pathway in spleen. **(B)** Structural features of MHCs and antigen peptide transporters.

In the spleen, almost all DEGs in antigen processing and presentation pathway were significantly upregulated at 12 h, especially the major histocompatibility complexes (MHC) including MHC_I–related gene protein (Mr1), MHC_I receptor (H2-k1), and MHC_II beta 1 chain (RT1-B) and transporters associated with antigen processing (TAP1 and TAP2) (**Figure 6A**). The structure predictions of DEGs showed that Mr1 and H2-k1 both contained an MHC_I domain, and RT1-B contained an MHC_II beta domain, besides, Mr1 and RT1-B both had an immunoglobulin C-Type (IGc1) domain and a transmembrane region. The TAP1 and TAP2 both contained a typical ABC_membrane domain and a AAA domain, while TAP1 had three transmembrane domains, and TAP2 had two transmembrane domains (**Figure 6B**).

### Reactive modules and hub genes in response to *V. harveyi* infection

**Figure 7:**
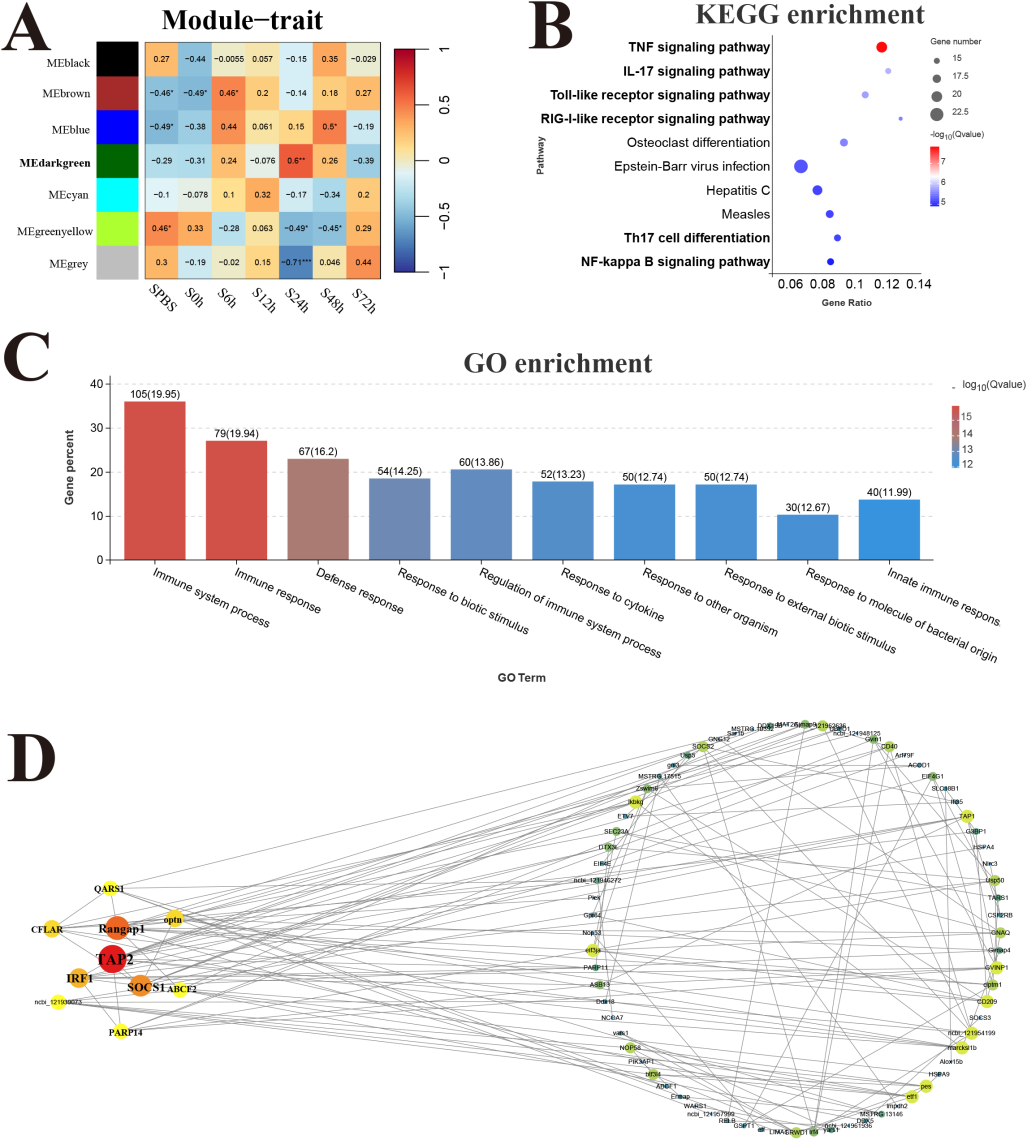
Weighted gene co-expression network analysis (WGCNA) of spleen. **(A)** Heat map of correlation between infection time and modules. **(B)** Enrichment pathway of dark green module. **(C)** GO enrichment of dark green module. **(D)** Gene co-expression network in the dark green module.

The response modules and hub genes of *P. leopardus* induced by *V. harveyi* infection were identified through the construction of a network using transcriptome datasets of spleen and liver. In the spleen, a total of seven modules were identified based on the gene expression patterns, and the dark green module with the strongest positive correlation (*r* = 0.6) was considered as a response module and included in subsequent analysis (**Figure 7A**). KEGG and GO enrichment analysis was performed after combining the genes from dark green module. Among the top 10 significantly enriched pathways, six pathways were related to immune function, namely, TNF signaling pathway, IL-17 signaling pathway, TLR signaling pathway, RIG-I receptor signaling pathway, Th17 cell differentiation, and NF-kappa B signaling pathway (**Figure 7B**). The GO analysis detected the significant enrichment associated with immune response (**Figure 7C**). Hub genes were identified according to the node connection degree that was significantly correlated with proximity to the center of the network. A total of 200 top genes with high connection degree were selected from dark green module, and the gene network diagram was constructed. The key hub genes were involved in immune regulation, including TAP2, interferon-related regulator 1 (IRF1), suppressor of cytokine signaling 1 (SOCS1), and CASP8 and FADD-like apoptosis regulator (CFLAR) (**Figure 7D**).

**Figure 8:**
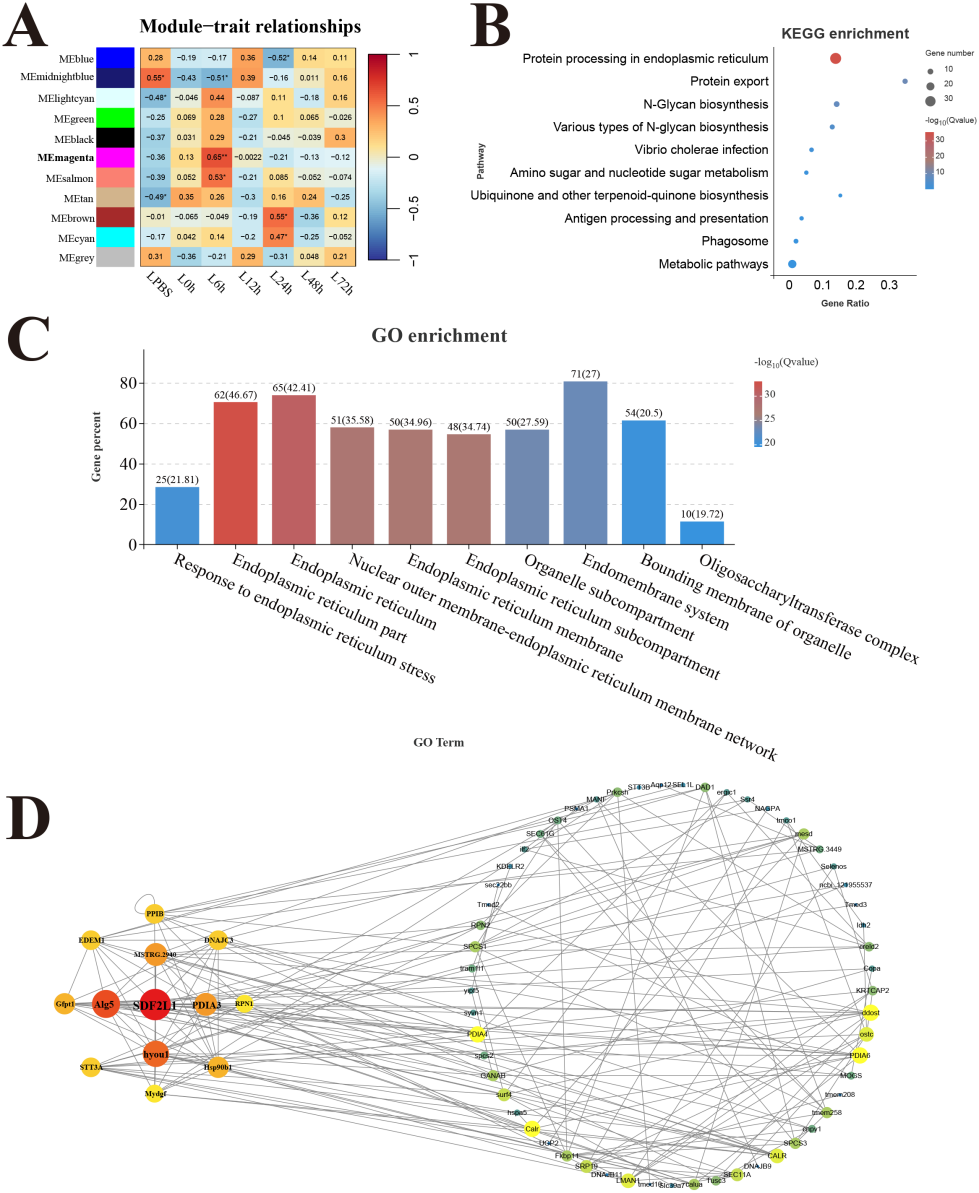
Weighted gene co-expression network analysis (WGCNA) of liver. **(A)** Heat map of correlation between infection time and modules. **(B)** Enrichment pathway of magenta module. **(C)** GO enrichment of magenta module. **(D)** Gene co-expression network in the magenta module.

In the liver, 11 modules were detected in the analysis, and the correlation heat map showed significant the magenta module with the strongest positive correlation (*r* = 0.65) was considered as a response module associated with L6h (**Figure 8A**). KEGG results revealed that genes in the magenta module were mainly enriched in the pathways related to protein process and biosynthesis, only the antigen processing and presentation pathway was involved in immune function (**Figure 8B**). GO enrichment results showed that genes in the magenta module were mainly related to the composition and process of endoplasmic reticulum (**Figure 8C**). Moreover, the key hub genes were stromal cell-derived factor 2-like protein 1 (SDF2L1), dolichyl-phosphate beta-glucosyltransferase (Alg5), protein disulfide-isomerase A3 (PDIA3), and so forth.

### qRT-PCR validation of immune genes

**Figure 9:**
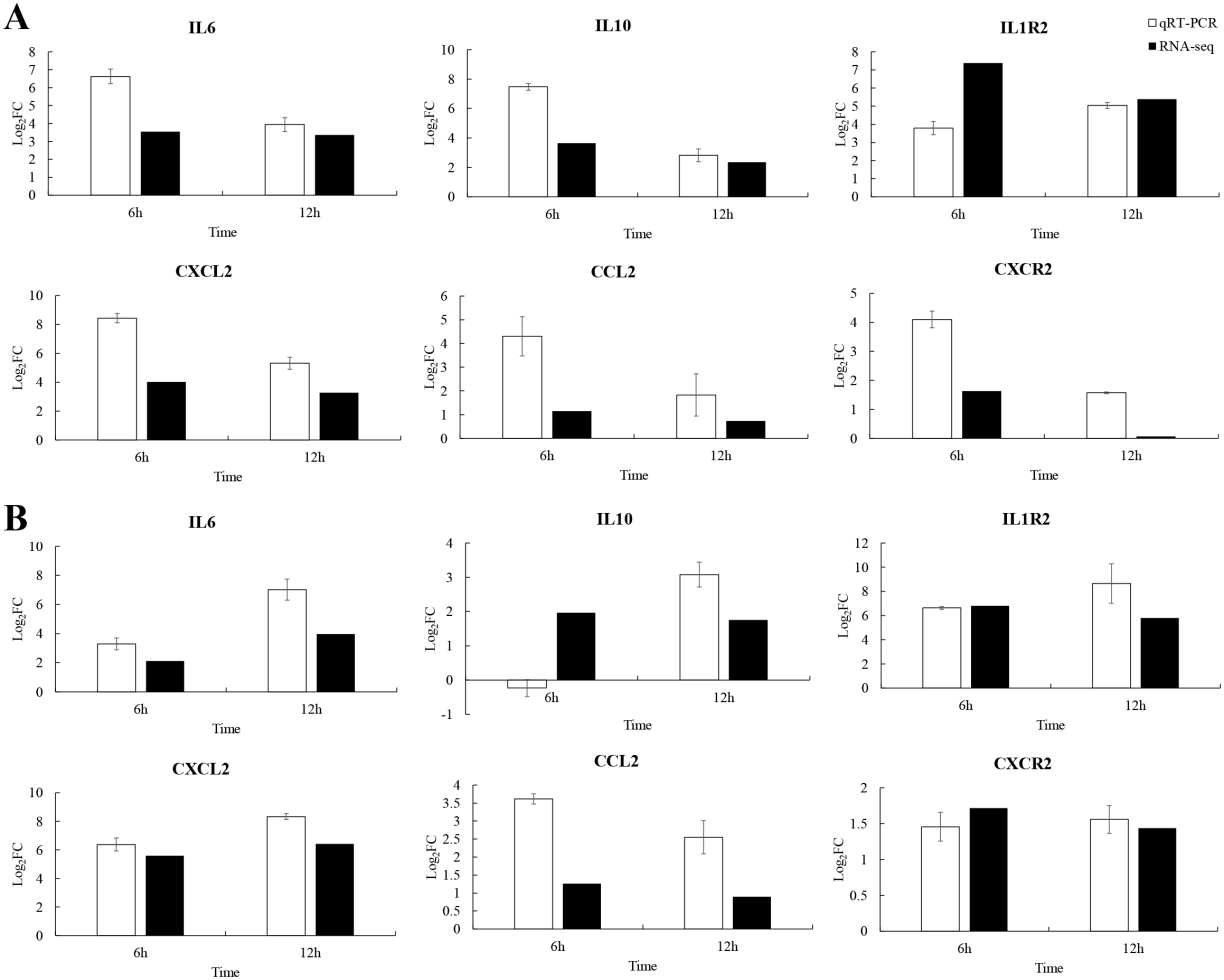
Comparison of qRT-PCR and RNA-seq for six DEGs at 6 and 12 h post-infection. Data shown are the mean of triplicates ± SD. **(A)** Spleen, **(B)** liver.

To explore the immune strategy of *P. leopardus* against *V. harveyi* infection, six immune-related genes were selected for qRT-PCR validation. Due to 6–12 h was very critical time points for *P. leopardus* against *V. harveyi* infection, 6 and 12 h were selected to detect the expression of immune-related genes. The results showed that the genes IL-6, IL1R2, CXCL2, CCL2, and CXCR2 associated with the cytokine–cytokine receptor interaction pathway in the spleen and liver, were significantly upregulated at 6 and 12 h, respectively. The IL-10 was significantly upregulated in the spleen at 6 and 12 h and in the liver at 12 h, while slightly downregulated in the liver at 6 h (**Figure 9**). These data showed that the results of qRT-PCR were broadly in line with the results of RNA-seq, indicating the accuracy of RNA-seq expression analysis.

## Discussion

*P. leopardus* is an economically important tropical fish owing to its bright body color, good taste, and high economic value. However, the rapid spread of *V. harveyi* has hindered the long-term healthy development of its aquaculture (21). A comprehensive understanding of the immune response of *P. leopardus* infected with *V. harveyi* may provide new ideas for disease prevention and treatment. The current study is the first to describe the transcriptome response of *P. leopardus* to *V. harveyi* at a whole infection stage.

### Dynamic immune process of spleen and liver in *P. leopardus* against *V. harveyi* infection

In previous research, transcriptome analysis of the immune response against bacterial infection were reported in a certain tissue or at a certain time point post-infection (24–26). However, the researches on the time course and tissue-specific immune response against bacterial infection were necessary to further explore the molecular mechanism of immune defense against bacterial infection in fish. In this study, we used RNA-seq to investigate the dynamic immune response against *V. harveyi* in the spleen and liver of *P. leopardus* at different time points post-infection. Compared to other infection time points, 6–12 h exhibited most DEGs in both spleen and liver, indicating that large-scale transcriptional alterations of the host genes occurred at the early infection stage. In addition, the KEGG enrichment analysis revealed that the innate immune related pathways were of significantly upregulated expression in both spleen and liver at the early infection stage. These results suggested that 6–12 h might be very critical time points for *P. leopardus* against *V. harveyi* infection. Similarly, rapid upregulation of innate immune signaling pathways was observed in *Cynoglossus semilaevis* against *V. harveyi* infection, indicating that treatment measures should be taken in the early stage after infection (27). Afterwards, the upregulated DEGs in the spleen were significantly enriched in the pathways related to cellular processes and DNA repair, which indicated that the spleen began to repair after completing its immune defense function. Based on the KEGG enrichment results, *V. harveyi* infection induced a dramatic expression decrease of genes related to lipid metabolism, amino acid metabolism and carbohydrate metabolism pathways in the liver at 6–48 h, which indicated that the metabolic function of liver was inhibited for the infection of *V. harveyi* at the early infection stage and middle infection stage. Intriguingly, some reports disclosed that the suppression of metabolic activities in host is possibly derived by host immune system (13). Keeping the immune-metabolic homeostasis is crucial for fish to adapt the ever-changing internal and external environment (28). As sufficient energy reserves are required to maintain the immune function and activate defense responses, it is speculated that when *P. leopardus* are infected by *V. harveyi*, the metabolic function of the liver needs to give way to the immune function to ensure adequate energy supply.

### Immune-related pathways of *P. leopardus* infected with *V. harveyi*

KEGG enrichment analysis identified key immune pathways required for antimicrobial infection, and the major pathways are discussed below. The inflammatory response consists of a cascade of highly orchestrated innate immunity events that occur upon recognition of pathogen associated molecules by the PRRs on fish’s resident immune cells (29). Once inflammatory processes are triggered, large amounts of pro-inflammatory cytokines are released, such as TNFs, ILs and interferons (IFNs) (30). In the present study, DEGs were mapped to many classical innate immune pathways, such as RIG-I-like receptor signaling pathway, TLR signaling pathway, IL-17 signaling pathway, and NOD-like receptor signaling pathway at the early infection stage in both spleen and liver. Tlr5, as a member of TLRs family in mammals, is responsible for recognizing bacterial flagellin and initiating innate immunity (31). In *Sinocyclocheilus graham*, two TLR5 genes (*sgTLR5a* and *sgTLR5b*) were upregulated notably in the liver, spleen, and head kidney tissues after stimulations of *Aeromonas hydrophila* and flagellin, and could positively regulated the NF-κB signaling pathway (32). In Nile tilapia, *OnTLR5* significantly upregulated *OnMyd88*-induced NF-κB activation and had an interaction with *Aeromonas flagellin* (33). NOD2 has been identified as an important sensor for microorganic invasion in both mammals and teleost fishes. In the teleost fish *Schizothorax prenanti*, NOD2 and its two splicing variants positively responded to exposure of *A. hydrophila* and LPS stimulation in varying degrees and could activate the NF-κB signal (34). NLRP3 inflammasome can be activated by a variety of stimuli and plays an important role in protecting host from pathogen invasion and maintaining homeostasis. In common carp, NLRP3 was significantly increased after stimulation with *E. tarda* and *A. hydrophila* and could form inflammasome through ASC-independent pathway (35). In *P. leopardus*, TLR5, NOD2, and NLRP3 were all notably induced in the spleen and liver from 6 to 12 h or 24 h post-infection, and the predicted protein structures of three genes were similarly to those of mammals or other fishes. The adaptor molecules can trigger downstream signaling pathways to drive the induction of proinflammatory cytokines (36). In this study, three adaptor molecules, including TRAF2, TRAF3, and IRAK4 with a variety of conserved structures like other species, participated into more than two innate immune receptor pathways of *P. leopardus* (37–39). The significantly upregulated expressions of these genes in *P. leopardus* at 6–12 h post-injection, indicated that they played important role in resistance to *V. harveyi*. Once inflammatory processes are triggered, large amounts of pro-inflammatory cytokines are activated and released (40). In mammals, TNF, IL-1b, and IL-6, as symbolic pro-inflammation cytokines, can activate macrophages and neutrophils directly, and IL-10 is an anti-inflammatory cytokine that mitigates exaggerated inflammatory immune reactions (41–43). In this study, TNFRSF11B, TNFRSF14, IL-1b, IL-6 and IL-10 in *P. leopardus* were upregulated significantly at 6–12 h after *V. harveyi* infection. Moreover, the predicted protein structures of these inflammatory cytokines contained their characteristic family domains. These results imply that inflammatory immune reaction of *P. leopardus* can be activated quickly to eliminate invading *V. harveyi*. IL-1b can bind to IL1R1 to activate the downstream signaling pathways, and IL1R2 lacking the TIR domain can bind fraudulently to IL-1b and prevent signal transduction (44, 45). In various fishes, IL1R1 and IL1R2 have been identified, and their mRNAs are notably induced after bacteria stimulation (46–48). In *P. leopardus*, the expression of IL1R1b and IL1R2 were significantly upregulated at 6–12 h post-infection. Similarly to other fishes, *P. leopardus* IL1Rs possess characteristic IG domains in the extracellular region and a cytoplasmic TIR domain except that IL1R2 lacks the TIR domain for signaling. These results reveal that inflammatory cytokines and their receptors in fish have similar protein structure and expression changes after bacterial infection, and the signal pathways of these inflammatory cytokines are conserved.

Chemokines are a family of cytokines that coordinate the movement or migration of immune cells from lymphoid organs to the site of action, playing a fundamental role for an efficient innate and adaptive immune response (49). Depending on the relative position of two N-terminal cysteine residues, chemokines are divided into five major subfamilies, including CC, CXC, CX3C, CX, and XC (only present in fish), in which CXC and CC are the dominant subfamilies (50). Up to now, many CC/CXC chemokine genes and their receptors have been identified in a number of fish species (51–53). In this study, chemokines (CCLs and CXCLs) and their receptors (CCRs and CXCRs) in *P. leopardus* also have a typical SCY structure and a Pfam 7tm_1 domain as other fishes, respectively. Moreover, CCL2, CCL21, CXCL2, CXCR2, and CXCR3 mRNAs were significantly induced at 6–12 h after *V. harveyi* infection, implying that *P. leopardus* immune cells can be activated and recruited to the sites of infection at early time points post-infection.

Antigen processing and presentation pathway is responsible for presenting pathogenic antigen to lymphocytes, and T-cell receptor participate in immunogenic antigen recognition peptide-MHC to activate and regulate humoral and cellular immune response (54). In teleost fishes, this pathway plays a necessary role in the adaptive immune response (55). In addition, transcriptome analyses of *Carassius auratus* and *Oreochromis niloticus* challenged by pathogens has revealed that antigen presentation signals were significantly enriched among post-infection altered genes (56, 57). The expression profiles identified MHC I antigen processing and presentation molecules, such as Mr1, H2-k1, TAP1/2, CANX, CALR, and HSP70, as well as MHC II-related genes, such as RT1-B and CTSL. In *P. leopardus*, MHC class I and MHC class II genes were all upregulated at 6–12 h after *V. harveyi* infection in this context, which were consistent with the finding in *Scophthalmus maximus*, indicating an important role of antigen processing and presentation in anti-bacterial infection (58, 59). Mr1 and H2-k1 consist of an MHC_I domain, and RT1-B had an MHC_II beta domain, which indicated that they were recognized as the classical MHC-related genes as other fishes (60, 61). TAP is a key component in the MHC class I–dependent antigen presentation pathway, which is primarily responsible for the transportation of peptides into the endoplasmic reticulum for MHC-I presentation. It is a member of the ABC [adenosine triphosphate (ATP)–binding cassette] family of transporters with conserved architecture of transmembrane domains, which translocate a wide range of substrates across membranes in an ATP-dependent manner (62). In *P. leopardus*, TAP1 and TAP2 were also found typical ABC_membrane domain and transmembrane domains. Taking together, the upregulation of MHC I and MHC II–associated genes indicates the efficient processing and presentation of *V. harveyi* antigens and thereby mounting of anti-*V. harveyi* response in *P. leopardus*.

### Key hub genes related to *V. harveyi* infection Identified by WGCNA

After analyzing WGCNA, it was found that immune system in the spleen of *P. leopardus* played crucial roles in responding to *V. harveyi* infection, providing valuable insights into the underlying molecular mechanisms. Additionally, this study identified four hub genes mainly involved in immune responses, including TAP2, IRF1, SOCS1, and CFLAR. The function of hub genes reflected the spleen function. TAP plays a key role in the MHC class I antigen presentation pathway (63). In fish, the characteristic and relationship with disease resistance of MHC I genes have been studied, but other genes in MHC I region were rarely reported. It was only reported that the TAP2 gene in grass carp were up regulated in the spleen and kidney after infection with *A. hydrophila* (64). In *P. leopardus*, the importance of TAP2 was emphasized by identified as hub gene with highly expressed in the spleen after *V. harveyi* infection. IRF1 is an important regulator in controlling the transcription of type I interferon genes, and its functions have been well characterized from lower vertebrates to higher vertebrates (65). Fish IRF1 of miiuy croaker (*Miichthys miiuy*) has been identified in previous study (66). Xuan et al. found that IRF1 negatively regulated NF-κB signaling by targeting MyD88 for degradation in teleost fish (67). In this study, IRF1 was significantly upregulated after *V. harveyi* infection, and its connection value was high in the co-expression module, suggesting its importance in the immunity regulation in the *P. leopardus*. The Socs proteins are crucial soluble mediators to inhibit signal transduction via the JAK-STAT pathway in the innate and adaptive immune responses, among which SOCS1 is the primary regulator of a number of cytokines (68). In salmon, the SOCS1 showed a strong negative regulatory activity on types I and II IFN signaling and the suppression of viral replication was partially rescued by over expression of SOCS1, which indicated that teleost SOCS-1 might also be involved in IFN signaling regulation (69, 70). CFLAR, also known ascFlip, plays a critical role in fundamental intracellular processes such as inflammation and apoptosis (71). As reported, CFLAR is a negative regulator of pathological cardiac remodeling and the resultant heart failure that was associated with its potential modulation of apoptosis, inflammation and fibrosis (72). Faiz et al. found that decreased CFLAR expression would increase the susceptibility of cell death (73). Given these intimate associations of CFLAR with inflammation and cell death, we supposed that CFLAR might have a potential role in regulating damage caused by infection with *V. harveyi*. Therefore, the hub genes TAP2, IRF1, SOCS1, and CFLAR identified based on WGCNA of spleen were significantly changed when the *P. leopardus* were infected by *V. harveyi*.

## Conclusion

**Figure 10:**
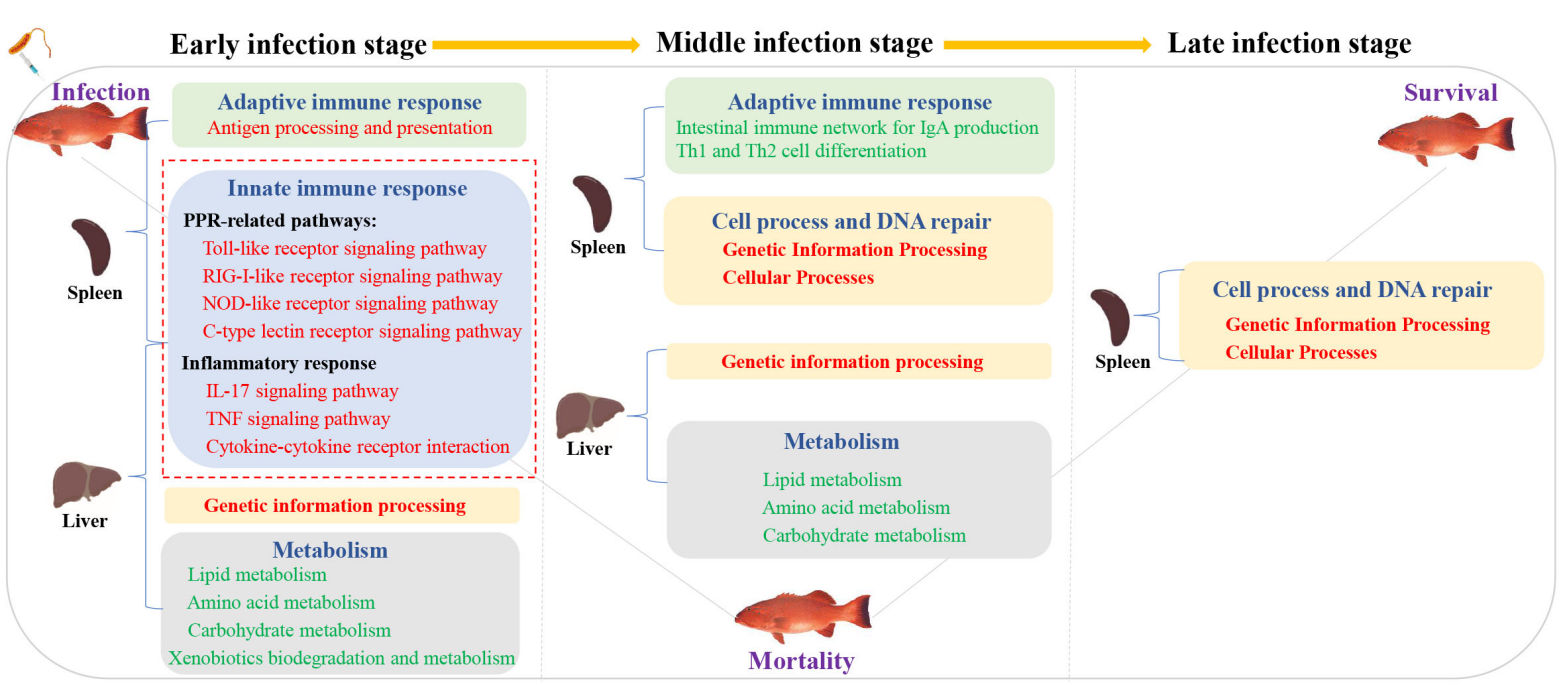
A hypothesis of the potential regulatory pathway during bacterial infection between the spleen and liver. Red font and green font represent upregulated and downregulated DEGs enrichment pathways, respectively. The red dotted box represents the key immune-related pathways focused on in this study.

To sum up, we provide the transcriptional spectrum of spleen and liver in *P. leopardus* at different time points after infection with *V. harveyi*. The defense responses of *P. leopardus* were characterized by a prevailing upregulation of innate immune pathways (e.g., PRR and inflammatory response related) in both spleen and liver and adaptive immune pathway (antigen processing and presentation) in the spleen during the early infection stage, and the concomitant the downregulation of metabolic activities in the liver until to the middle infection stage (**Figure 10**). The genes related to immune regulation including TAP2, IRF1, Socs1, and CFLAR were found to be hub genes of spleen in the gene co-expression network by WGCNA analysis. Our research provides important information to reveal the immune defense related pathways and genes of the *P. leopardus*, and also provides molecular data and targets for genetic improvement of resistance to bacterial disease in *P. leopardus*.

## Data Availability

The datasets presented in this study can be found in online repositories. The names of the repository/repositories and accession number(s) can be found below: https://www.ncbi.nlm.nih.gov/, PRJNA1126611.

## Supplementary material

Supplementary Figure 1 Veen diagrams representing differentially expressed genes (DEGs) in the spleen and liver at different time points. Red and blue circle representing the number of DEGs in the liver and spleen, respectively. Gray circle representing the number of common DEGs in the liver and spleen.

Supplementary Figure 2 The 10 most enriched GO terms in the spleen **(A)** and liver **(B)** of *P. leopardus* at different time points following *V. harveyi* challenge. I: biological process, II: molecular function, III: cellular component. The *y*-axis representing gene percent. The column color representing the significant degree of enrichment of GO term.

## References

[1] Zhou Q Lu S Liu Y Zhou B Chen SL. Development of a 20 K SNP array for the leopard coral grouper. Plectropomus leopardus. Aquaculture. (2024) 578:740079. doi: 10.1016/j.aquaculture.2023.740079

[2] Payet SD Lowe JR Mapstone BD Pratchett MS Sinclair-Taylor TH Taylor BM. Comparative demography of commercially important species of coral grouper, *Plectropomus leopardus* and P. laevis, from Australia's great barrier reef and Coral Sea marine parks. J Fish Biol. (2020) 97:1165–76. doi: 10.1111/jfb.14491 32785930

[3] Zhu XW Hao RJ Zhang JP Tian CX Hong YC Zhu CH. Dietary astaxanthin improves the antioxidant capacity, immunity and disease resistance of coral trout (*Plectropomus leopardus*). Fish Shellfish Immunol. (2022) 122:38–47. doi: 10.1016/j.fsi.2022.01.037 35085737

[4] Zhu XW Hao RJ Zhang JP Tian CX Hong YC Zhu CH. Improved growth performance, digestive ability, antioxidant capacity, immunity and *Vibrio harveyi* resistance in coral trout (*Plectropomus leopardus*) with dietary vitamin C. Aquacult Rep. (2022) 24:101111. doi: 10.1016/j.aqrep.2022.101111

[5] Wang L Liu Y Zhang ZW Li KM Zhou Q Zhang TS. Molecular characterization of immunoglobulin M (IgM) and polymeric immunoglobulin receptor (pIgR) and expression response to *Vibrio harveyi* challenge in leopard coral grouper (*Plectropomus leopardus*). Aquac Res. (2023) 2023(1):8883767. doi: 10.1155/2023/8883767

[6] Secombes C Wang T Hong S Peddie S Crampe M Laing K. Cytokines and innate immunity of fish. Dev Comp Immunol. (2001) 25:713–23. doi: 10.1016/S0145-305X(01)00032-5 11602192

[7] Qiao Y Ma XW Zhong SP Zhang M. Interaction analysis of miRNA and mRNA in the head kidney of black seabass (*Centropristis striata*) after *Vibrio harveyi* infection. Aquaculture. (2021) 542:736886. doi: 10.1016/j.aquaculture.2021.736886

[8] Wang J Wang SP Zhang JW Zhu CH Chen SL Zhou Q. Integrated transcriptomic and metabolomic analysis provides insights into the responses to Vibrio infection in *Plectropomus leopardus* . Aquaculture. (2024) 587:740854. doi: 10.1016/j.aquaculture.2024.740854

[9] Zhou ZY He SY Lu CW Bai SJ Kuang LF Yang B. *Nocardia seriolae* mediates granulomatous chronic inflammation of spleen in *Micropterus salmoides* through necroptosis. Aquaculture. (2024) 580:740360. doi: 10.1016/j.aquaculture.2023.740360

[10] Floreste FR Titon B Titon SCM Muxel SM Figueiredo ACD Gomes FR. Liver vs. spleen: Time course of organ-dependent immune gene expression in an LPS-stimulated toad (*Rhinella diptycha*). Comp Biochem Phys B. (2022) 263:110784. doi: 10.1016/j.cbpb.2022.110784 35931313

[11] Smith NC Rise ML Christian SL. A comparison of the innate and adaptive immune Systems in Cartilaginous Fish, ray-finned fish, and lobe-finned fish. Front Immunol. (2019) 10:2292. doi: 10.3389/fimmu.2019.02292 31649660 PMC6795676

[12] Wang F Zhong ZR Xie Q Ou J Xiong NX Huang MZ. Multiomics analyses explore the immunometabolic interplay in the liver of white crucian carp (*Carassius cuvieri*) after Aeromonas veronii challenge. Mar Biotech. (2024) 26:790–809. doi: 10.1007/s10126-024-10347-3 39042324

[13] Zmora N Bashiardes S Levy M Elinav E. The role of the immune system in metabolic health and disease. Cell Metab. (2017) 25:506–21. doi: 10.1016/j.cmet.2017.02.006 28273474

[14] Rauw WM. Immune response from a resource allocation perspective. Front Genet. (2012) 3:267–80. doi: 10.3389/fgene.2012.00267 PMC3571735 23413205

[15] Fonseca MT Moretti EH Marques LMM MaChado BF Brito CF Guedes JT. A leukotriene-dependent spleen-liver axis drives TNF production in systemic inflammation. Sci Signaling. (2021) 14:eabb0969. doi: 10.1126/scisignal.abb0969 33879603

[16] Liu QN Xin ZZ Liu Y Zhang DZ Jiang SH Chai XY. *De novo* transcriptome assembly and analysis of differential gene expression following lipopolysaccharide challenge in *Pelteobagrus fulvidraco* . Fish Shellfish Immunol. (2018) 73:84–91. doi: 10.1016/j.fsi.2017.11.045 29191796

[17] Zou M Zhang XT Shi ZC Lin L Ouyang G Zhang GR. A comparative transcriptome analysis between wild and albino yellow catfish (*Pelteobagrus fulvidraco*). PloS One. (2015) 10(6):e0131504. doi: 10.1371/journal.pone.0131504 26114548 PMC4482592

[18] Ye H Xiao SJ Wang XQ Wang ZY Zhang ZS Zhu CK. Characterization of Spleen Transcriptome of *Schizothorax prenanti* during *Aeromonas hydrophila* Infection. Mar Biotechnol. (2018) 20:246–56. doi: 10.1007/s10126-018-9801-0 29516376

[19] Baliarsingh S Chung JM Sahoo S Sarkar A Mohanty J Han YS. Transcriptome analysis of *Macrobrachium rosenbergii* hepatopancreas in response to *Vibrio harveyi* infection. Aquac Res. (2021) 52:1855–75. doi: 10.1111/are.15034

[20] Xiu YJ Zhang HX Wang SY Gan T Wei M Zhou S. cDNA cloning, characterization, and expression analysis of the Rac1 and Rac2 genes from *Cynoglossus semilaevis* . . Fish Shellfish Immunol. (2019) 84:998–1006. doi: 10.1016/j.fsi.2018.11.006 30399403

[21] Lu S Liu Y Qu SY Zhou Q Wang L Zhang TS. Genomic prediction of survival against Vibrio harveyi in leopard coral grouper (Plectropomus leopardus) using GBLUP, weighted GBLUP, and BayesCπ. Aquaculture. (2023) 572:739536. doi: 10.1016/j.aquaculture.2023.739536

[22] Zhou Q Guo X Huang Y Gao H Xu H Liu S. *De novo* sequencing and chromosomal-scale genome assembly of leopard coral grouper, *Plectropomus leopardus* . Mol Ecol Resour. (2020) 20:1403–13. doi: 10.1111/1755-0998.13207 32521104

[23] Almeida-Silva F Venancio TM. BioNERO: an all-in-one R/Bioconductor package for comprehensive and easy biological network reconstruction. Funct Integr Genomics. (2022) 22:131–6. doi: 10.1007/s10142-021-00821-9 34787733

[24] Zhao X Zhang Y Gao TX Song N. Spleen transcriptome profiling reveals divergent immune responses to LPS and Poly (I:C) challenge in the yellow drum (*Nibea albiflora*). Int J Mol Sci. (2023) 24(9):7735. doi: 10.3390/ijms24097735 37175446 PMC10178140

[25] Diao J Yu XQ Wang XL Fan Y Wang SX Li L. Full-length transcriptome sequencing combined with RNA-seq analysis revealed the immune response of fat greenling (*Hexagrammos otakii*) to *Vibrio harvey* in early infection. Microb Pathogenesis. (2020) 149:104527. doi: 10.1016/j.micpath.2020.104527 32980468

[26] Gnanagobal H Chakraborty S Vasquez I Chukwu-Osazuwa J Cao T Hossain A. Transcriptome profiling of lumpfish (*Cyclopterus lumpus*) head kidney to *Renibacterium salmoninarum* at early and chronic infection stages. Dev Comp Immunol. (2024) 156:105165. doi: 10.1016/j.dci.2024.105165 38499166

[27] Zhang XH Hao XC Ma WX Zhu TF Zhang ZH Wang Q. Transcriptome Analysis Indicates Immune Responses against *Vibrio harveyi* in Chinese Tongue Sole (*Cynoglossus semilaevis*). Animals. (2022) 12:1144. doi: 10.3390/ani12091144 35565570 PMC9104532

[28] Zhao Y Weng M Zhang Q Li A Zhang J. Transcriptomics analysis of the infected tissue of gibel carp (*Carassius auratus gibelio*) with liver myxobolosis infers the underlying defense mechanisms from the perspective of immune-metabolic interactions. Aquaculture. (2021) 542:736867. doi: 10.1016/j.aquaculture.2021.736867

[29] Floreste FR Titon B Titon SCM Muxel SM Figueiredo ACD Gomes FR. Liver vs. spleen: Time course of organ-dependent immune gene expression in an LPS-stimulated toad (*Rhinella diptycha*). Comp Biochem Phys B. (2023) 263:110784. doi: 10.1016/j.cbpb.2022.110784 35931313

[30] Sakai M Hikima J Kono T. Fish cytokines: current research and applications. Fisheries Sci. (2021) 87:1–9. doi: 10.1007/s12562-020-01476-4

[31] Kawai T Akira SJNI. The role of pattern-recognition receptors in innate immunity: update on Toll-like receptors. Nat Immunol. (2010) 11:373. doi: 10.1038/ni.1863 20404851

[32] Yang SY Leng SZ Li YK Wang XA Zhang YW Wu AL. Identification and functional characteristics of two TLR5 subtypes in S. grahami. Fish Shellfish Immunol. (2022) 131:707–17. doi: 10.1016/j.fsi.2022.10.041 36309325

[33] Gao FY Pang JC Lu MX Liu ZG Wang M Ke XL. TLR5 recognizes *Aeromonas hydrophila* flagellin and interacts with MyD88 in *Nile tilapia* . Dev Comp Immunol. (2022) 133:104409. doi: 10.1016/j.dci.2022.104409 35405183

[34] Li YK Jin L Xia PZ Sui WK Huang AQ Bu GX. Identification and functional analysis of NOD2 and its two splicing variants associated with a novel pattern of signal regulation in teleost fishes. Dev Comp Immunol. (2021) 120:104049. doi: 10.1016/j.dci.2021.104049 33609614

[35] Zhao Y Liang YX Chen QH Shan SJ Yang GW Li H. The function of NLRP3 in anti-infection immunity and inflammasome assembly of common carp (*Cyprinus carpio* L.). Fish Shellfish Immunol. (2024) 145:109367. doi: 10.1016/j.fsi.2024.109367 38211703

[36] Wang KL Chen SN Li L Huo HJ Nie P. Functional characterization of four TIR domain-containing adaptors, MyD88, TRIF, MAL, and SARM in mandarin fish Siniperca chuatsi. Dev Comp Immunol. (2021) 122:104110. doi: 10.1016/j.dci.2021.104110 33933533

[37] Kim WL Kim MS Kim KH. Molecular cloning of rock bream's (*Oplegnathus fasciatus*) tumor necrosis factor receptor-associated factor 2 and its role in NF-κB activiation. Fish Shellfish Immunol. (2011) 30:1178–83. doi: 10.1016/j.fsi.2011.02.007 21320605

[38] Xu YP Zhou YL Xiao Y Gu WB Li B Cheng YX. Functional differences in the products of two TRAF3 genes in antiviral responses in the Chinese giant salamander, *Andrias davidianus* . Dev Comp Immunol. (2021) 119:104015. doi: 10.1016/j.dci.2021.104015 33460679

[39] Han XQ Gao FY Lu MX Liu ZG Wang M Ke XL. Molecular characterization, expression and functional analysis of IRAK1 and, Cheek for IRAK4 in Nile tilapia (*Oreochromis niloticus*). Fish Shellfish Immunol. (2020) 97:135–45. doi: 10.1016/j.fsi.2019.12.041 31846774

[40] Netea MG Balkwill F Chonchol M Cominelli F Donath MY Giamarellos-Bourboulis EJ. A guiding map for inflammation. Nat Immunol. (2017) 18:826–31. doi: 10.1038/ni.3790 PMC5939996 28722720

[41] Dinarello CA. Overview of the IL-1 family in innate inflammation and acquired immunity. Immunol Rev. (2018) 281:8–27. doi: 10.1111/imr.12621 29247995 PMC5756628

[42] Secombes CJ Wang T Bird S. The interleukins of fish. Dev Comp Immunol. (2011) 35:1336–45. doi: 10.1016/j.dci.2011.05.001 21605591

[43] Wang LQ Liu CY He HX Chen JP He X Qin QW. Largemouth bass Rel exerts antiviral role against fish virus and regulates the expression of interleukin-10. Fish Shellfish Immunol. (2023) 142:109117. doi: 10.1016/j.fsi.2023.109117 37778738

[44] Deng YD Ding CH Yang H Zhang MY Xiao Y Wang HQ. First in *vitro* and in *vivo* evaluation of recombinant IL-1β protein as a potential immunomodulator against viral infection in fish. Int J Biol Macromol. (2024) 255:128192. doi: 10.1016/j.ijbiomac.2023.128192 37979760

[45] Zhou N Chen L-L Chen J Guo Z-P. Molecular characterization and expression analysis of IL-1β and two types of IL-1 receptor in barbel steed (*Hemibarbus labeo*). Comp Biochem Phys B. (2020) 241:110393. doi: 10.1016/j.cbpb.2019.110393 31866568

[46] Gao JD Jiang XY Wang JY Xue YJ Li X Sun ZS. Phylogeny and expression modulation of interleukin 1 receptors in grass carp (*Ctenopharyngodon idella*). Dev Comp Immunol. (2019) 99:103401. doi: 10.1016/j.dci.2019.103401 31145914

[47] Eggestol HO Lunde HS Knutsen TM Haugland GT. Interleukin-1 ligands and receptors in lumpfish (*Cyclopterus lumpus* L.): Molecular characterization, phylogeny, gene expression, and transcriptome analyses. Front Immunol. (2020) 11:502. doi: 10.3389/fimmu.2020.00502 32300342 PMC7144542

[48] Zhang XS Xu S Lu WQ. Interleukin 1 receptor type I (IL-1RI) is involved in the innate immune response of olive flounder (*Paralichthys olivaceus*) to resist pathogens. Fish Shellfish Immunol. (2021) 119:51–9. doi: 10.1016/j.fsi.2021.09.020 34592473

[49] Valdés N Cortés M Barraza F Reyes-López FE Imarai M. CXCL9-11 chemokines and CXCR3 receptor in teleost fish species. Fish Shellfish Immunol Rep. (2022) 3:100068. doi: 10.1016/j.fsirep.2022.100068 36569039 PMC9782732

[50] Zhou S Mu Y Liu Y Ao J Chen X. Identification of a fish specific chemokine CXCL_F2 in large yellow croaker (*Larimichthys crocea*) reveals its primitive chemotactic function. Fish Shellfish Immunol. (2016) 59:115–22. doi: 10.1016/j.fsi.2016.10.012 27729274

[51] Fu Q Li YQ Zhao SC Cao M Yang N Huo HJ. CC chemokines and their receptors in black rockfish (*Sebastes schlegelii*): Characterization, evolutionary analysis, and expression patterns after *Aeromonas Salmonicida* infection. Aquaculture. (2022) 546:737377. doi: 10.1016/j.aquaculture.2021.737377

[52] Kim JW Kim EG Kim DH Shim SH Park CI. Molecular identification and expression analysis of the CC chemokine gene in rock bream (*Oplegnathus fasciatus*) and the biological activity of the recombinant protein. Fish Shellfish Immunol. (2013) 34:892–901. doi: 10.1016/j.fsi.2012.12.013 23357024

[53] Oehlers SHB Flores MV Hall CJ O'Toole R Swift S Crosier KE. Expression of zebrafish cxcl8 (interleukin-8) and its receptors during development and in response to immune stimulation. Dev Comp Immunol. (2010) 34:352–9. doi: 10.1016/j.dci.2009.11.007 19941893

[54] Teng J Zhao Y Meng QL Zhu SR Chen HJ Xue LY. Transcriptome analysis in the spleen of Northern Snakehead (*Channa argus*) challenged with Nocardia seriolae. Genomics. (2022) 114:110357. doi: 10.1016/j.ygeno.2022.110357 35378240

[55] Zhao Y Liu X Sato H Zhang Q Li A Zhang J. RNA-seq analysis of local tissue of *Carassius auratus gibelio* with pharyngeal myxobolosis: Insights into the pharyngeal mucosal immune response in a fish-parasite dialogue. Fish Shellfish Immunol. (2019) 94:99–112. doi: 10.1016/j.fsi.2019.08.076 31476388

[56] Hu X Bai J Liu R Lv A. Comprehensive transcriptomics and proteomics analysis of *Carassius auratus* gills in response to *Aeromonas hydrophila* . Fish Shellfish Immunol Rep. (2023) 4:100077. doi: 10.1016/j.fsirep.2022.100077 36589261 PMC9798182

[57] Sood N Verma DK Paria A Yadav SC Yadav MK Bedekar MK. Transcriptome analysis of liver elucidates key immune-related pathways in Nile tilapia *Oreochromis niloticus* following infection with tilapia lake virus. Fish Shellfish Immunol. (2021) 111:208–19. doi: 10.1016/j.fsi.2021.02.005 33577877

[58] Zhang J Zhang S Sun X Xu X. Comparative transcriptome analysis reveals the immune response of turbot (*Scophthalmus maximus*) induced by inactivated bivalent vaccine. Fish Shellfish Immunol. (2023) 132:108461. doi: 10.1016/j.fsi.2022.108461 36462744

[59] Wang B Du H-H Huang H-Q Xian J-A Xia Z-H Hu Y-H. Major histocompatibility complex class I (MHC Iα) of Japanese flounder (*Paralichthys olivaceus*) plays a critical role in defense against intracellular pathogen infection. Fish Shellfish Immunol. (2019) 94:122–31. doi: 10.1016/j.fsi.2019.09.005 31491527

[60] Pinto RD Randelli E Buonocore F Pereira PJB dos Santos NMS. Molecular cloning and characterization of sea bass (*Dicentrarchus labrax*, L.) MHC class I heavy chain and β2-microglobulin. Dev Comp Immunol. (2013) 39:234–54. doi: 10.1016/j.dci.2012.10.002 23116964

[61] Cao ZJ He MW Chen XJ Wang SF Cai Y Xie ZY. Identification, polymorphism and expression of MHC class Iα in golden pompano, *Trachinotus ovatus* . Fish Shellfish Immunol. (2017) 67:55–65. doi: 10.1016/j.fsi.2017.05.058 28554837

[62] Pinto RD Pereira PJB dos Santos NMS. Transporters associated with antigen processing (TAP) in sea bass (*Dicentrarchus labrax*, L.): Molecular cloning and characterization of TAP1 and TAP2. Dev Comp Immunol. (2011) 35:1173–81. doi: 10.1016/j.dci.2011.03.024 21540052

[63] Eggensperger S Tampé R. The transporter associated with antigen processing: a key player in adaptive immunity. Biol Chem. (2015) 396:1059–72. doi: 10.1515/hsz-2014-0320 25781678

[64] Bracamonte SE Smith S Hammer M Pavey SA Sunnucks P Beheregaray LB. Characterization of MHC class IIB for four endangered Australian freshwater fishes obtained from ecologically divergent populations. Fish Shellfish Immunol. (2015) 46:468–76. doi: 10.1016/j.fsi.2015.06.009 26093210

[65] Gan Z Cheng J Hou J Xia LQ Lu YS Nie P. Molecular and functional characterization of interferon regulatory factor 1 (IRF1) in amphibian *Xenopus tropicalis* . Int J Biol Macromol. (2021) 167:719–25. doi: 10.1016/j.ijbiomac.2020.11.217 33279564

[66] Shu C Sun YY Xu TJ. Molecular characterization of three IRF1 subfamily members reveals evolutionary significance of IRF11 in miiuy croaker. Dev Comp Immunol. (2015) 53:385–91. doi: 10.1016/j.dci.2015.07.009 26187301

[67] Xuan M Yan X Liu X Xu T. IRF1 negatively regulates NF-κB signaling by targeting MyD88 for degradation in teleost fish. Dev Comp Immunol. (2020) 110:103709. doi: 10.1016/j.dci.2020.103709 32348788

[68] Wang G Liu W Wang C Wang J Liu H Hao D. Molecular characterization and immunoregulatory analysis of suppressors of cytokine signaling 1 (SOCS1) in black rockfish, *Sebastes schlegeli* . Dev Comp Immunol. (2022) 130:104355. doi: 10.1016/j.dci.2022.104355 35077723

[69] Skjesol A Liebe T Iliev DB Thomassen EIS Tollersrud LG Sobhkhez M. Functional conservation of suppressors of cytokine signaling proteins between teleosts and mammals: Atlantic salmon SOCS1 binds to JAK/STAT family members and suppresses type I and II IFN signaling. Dev Comp Immunol. (2014) 45:177–89. doi: 10.1016/j.dci.2014.02.009 24582990

[70] Sobhkhez M Joensen LL Tollersrud LG Strandskog G Thim HL Jorgensen JB. A conserved inhibitory role of suppressor of cytokine signaling 1 (SOCS1) in salmon antiviral immunity. Dev Comp Immunol. (2017) 67:66–76. doi: 10.1016/j.dci.2016.11.001 27818171

[71] Wang XH Zhao J Guo HM Fan QQ. CFLAR is a critical regulator of cerebral ischaemia-reperfusion injury through regulating inflammation and endoplasmic reticulum (ER) stress. BioMed Pharmacother. (2019) 117:109155. doi: 10.1016/j.biopha.2019.109155 31387178

[72] Gehrke N Garcia-Bardon D Mann A SChad A Alt Y Wörns MA. Acute organ failure following the loss of anti-apoptotic cellular FLICE-inhibitory protein involves activation of innate immune receptors. Cell Death Differ. (2015) 22:826–37. doi: 10.1038/cdd.2014.178 PMC4392079 25342470

[73] Faiz A Heijink IH Vermeulen CJ Guryev V van den Berge M Nawijn MC. Cigarette smoke exposure decreases CFLAR expression in the bronchial epithelium, augmenting susceptibility for lung epithelial cell death and DAMP release. Sci Rep. (2018) 8(1):12426. doi: 10.1038/s41598-018-30602-7 30127367 PMC6102306

