# Supplementary figures and images for: The dynamic immune response of the liver and spleen in leopard coral grouper (Plectropomus leopardus) to Vibrio harveyi infection based on transcriptome analysis
Source: Front Immunol. 2024 Oct 10;15:1457745. doi: 10.3389/fimmu.2024.1457745 (PMC11499110; doi:10.3389/fimmu.2024.1457745)

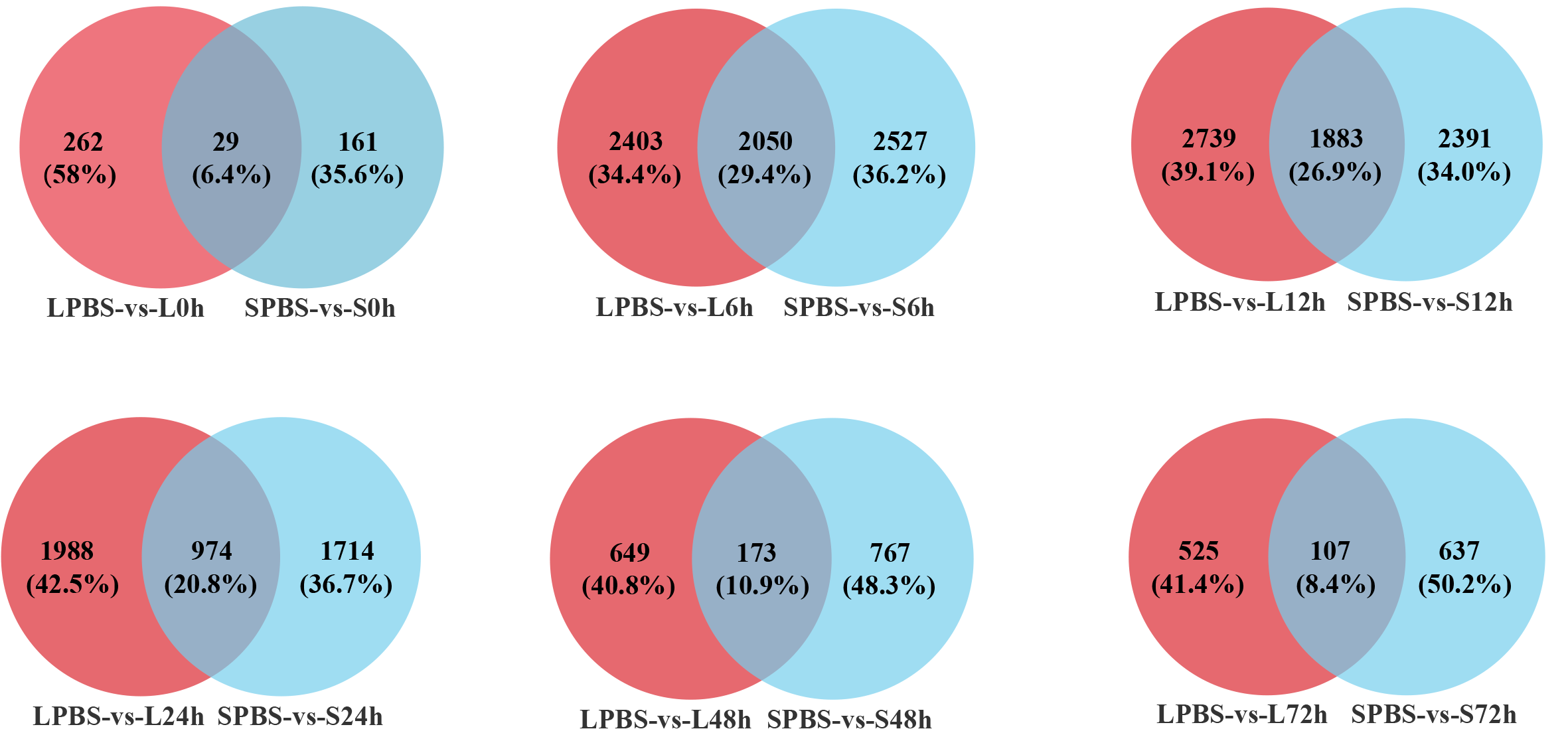

Supplement: Supplementary Figure 1 — Veen diagrams representing differentially expressed genes (DEGs) in the spleen and liver at different time points. Red and blue circle representing the number of DEGs in the liver and spleen, respectively. Gray circle representing the number of common DEGs in the liver and spleen. [file Image1.tif]

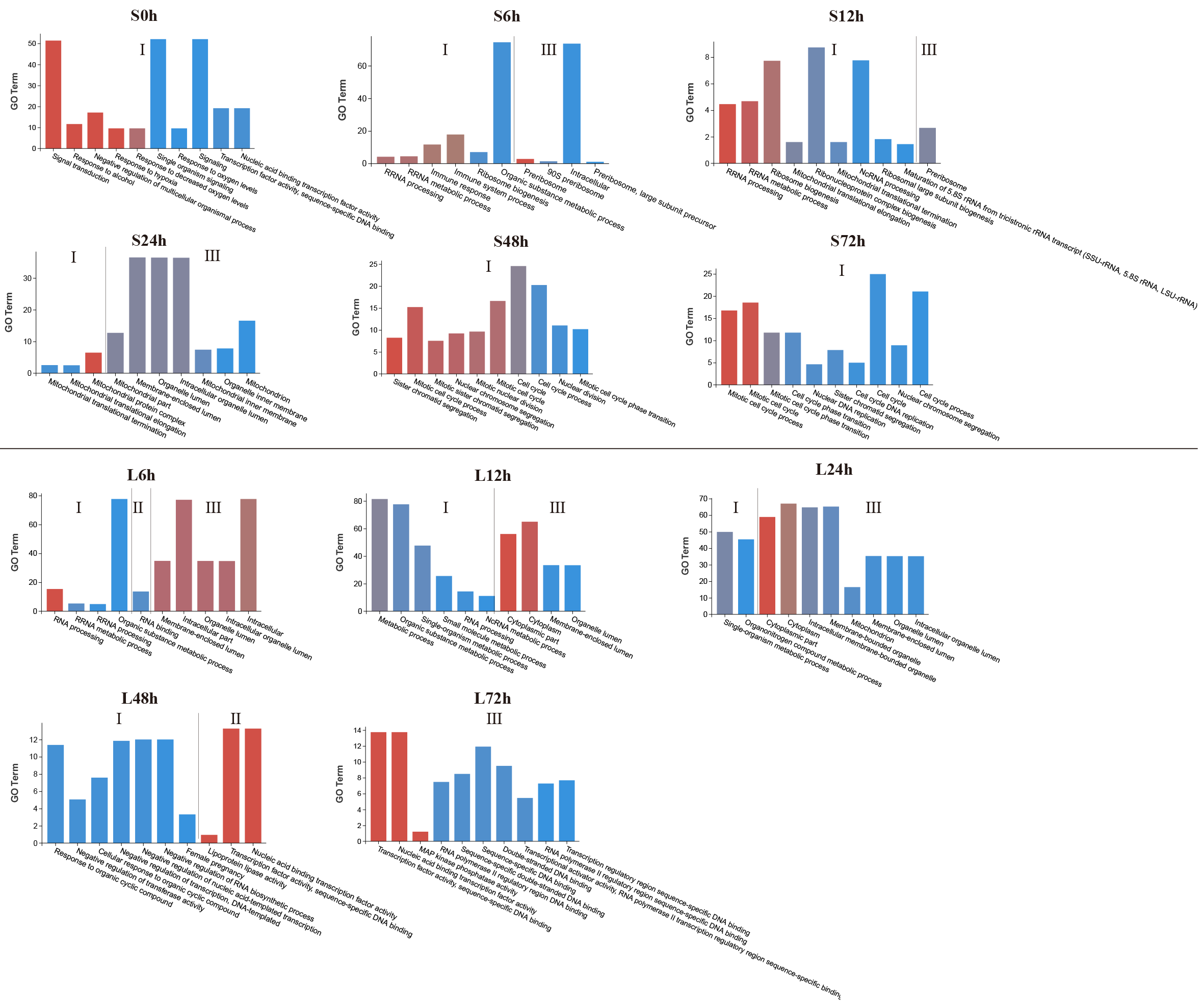

Supplement: Supplementary Figure 2 — The 10 most enriched GO terms in the spleen (A) and liver (B) of P. leopardus at different time points following V. harveyi challenge. I: biological process, II: molecular function, III: cellular component. The y-axis representing gene percent. The column color representing the significant degree of enrichment of GO term. [file Image2.tif]
