# Supplementary material for: The dynamic immune response of the liver and spleen in leopard coral grouper (Plectropomus leopardus) to Vibrio harveyi infection based on transcriptome analysis
Source: Front Immunol. 2024 Oct 10;15:1457745. doi: 10.3389/fimmu.2024.1457745 (PMC11499110; doi:10.3389/fimmu.2024.1457745)
Supplement: Supplementary file 3 [file DataSheet1.pdf]

Table S1 Sequences of the primers used for gene expression analysis by qRT-PCR

Gene Symbol	Forward primer (5′–3′)	Reverse primer (5′–3′)	Product size (bp)
IL6	TGCTCAGAGGTCAGACGCTTCAG	TCCTGGCTGCTGGTCACTGC	86
IL10	CCTCCTGTCTGTCCTGGTTCTCC	CCTCCACGAAACGACAGCACTG	76
IL1R2	TCAGCGTGGACGGAGTGGAC	ATTCAGCGGCGAGACAATCACAG	132
CXCL2	GTGAAGCAAGCCTTGGAGTGGAG	AGTGGGAGTTGGGAGGAATCAGC	95
CCL2	GGTGGGATAGCCAGTTGTTGTCG	AGGGCACGGTGGTTTATGTTGTTC	81
CXCR2	ACAGCATCACCATCGCAAGGC	CAGCACAGCAGAAACGCAATCAC	89
