# Supplementary material for: The dynamic immune response of the liver and spleen in leopard coral grouper (Plectropomus leopardus) to Vibrio harveyi infection based on transcriptome analysis
Source: Front Immunol. 2024 Oct 10;15:1457745. doi: 10.3389/fimmu.2024.1457745 (PMC11499110; doi:10.3389/fimmu.2024.1457745)
Supplement: Supplementary file 4 [file DataSheet2.pdf]

Table S2 P value and Q value (Adjusted P value) after GO enrichment analysis

Tissue	Time	GO term	GO ID	Pvalue	Qvalue
Spleen	0 h	signal transduction	GO:0007165	5.09E-07	0.001215
		response to alcohol	GO:0097305	1.01E-06	0.001215
		negative regulation of multicellular organismal process	GO:0051241	1.32E-06	0.001215
		response to hypoxia	GO:0001666	1.43E-06	0.001215
		response to decreased oxygen levels	GO:0036293	2.04E-06	0.001389
		transcription factor activity, sequence-specific DNA binding	GO:0003700	5.00E-06	0.001807
		nucleic acid binding transcription factor activity	GO:0001071	5.39E-06	0.001807
		single organism signaling	GO:0044700	3.75E-06	0.001879
		response to oxygen levels	GO:0070482	4.35E-06	0.001879
		signaling	GO:0023052	4.42E-06	0.001879
	6 h	preribosome	GO:0030684	2.30E-26	3.27E-23
		rRNA processing	GO:0006364	1.31E-23	1.57E-19
		rRNA metabolic process	GO:0016072	3.29E-23	1.97E-19
		immune response	GO:0006955	1.41E-21	5.64E-18
		immune system process	GO:0002376	3.62E-21	1.09E-17
		90S preribosome	GO:0030686	2.08E-17	1.48E-14
		ribosome biogenesis	GO:0042254	7.31E-17	1.76E-13
		organic substance metabolic process	GO:0071704	4.57E-16	9.14E-13
		intracellular	GO:0005622	5.97E-15	2.83E-12
		preribosome, large subunit precursor	GO:0030687	1.48E-14	5.24E-12
	12 h	rRNA processing	GO:0006364	1.70E-25	1.93E-21
		rRNA metabolic process	GO:0016072	2.73E-24	1.55E-20
		ribosome biogenesis	GO:0042254	2.67E-22	1.01E-18
		preribosome	GO:0030684	9.23E-20	1.30E-16
		mitochondrial translational elongation	GO:0070125	5.56E-20	1.58E-16
		ribonucleoprotein complex biogenesis	GO:0022613	1.53E-19	3.49E-16
		mitochondrial translational termination	GO:0070126	4.92E-19	9.34E-16
		ncRNA processing	GO:0034470	1.81E-17	2.65E-14
		ribosomal large subunit biogenesis	GO:0042273	1.86E-17	2.65E-14
		maturation of 5.8S rRNA from tricistronic rRNA transcript (SSU-rRNA, 5.8S rRNA, LSU-rRNA)	GO:0000466	3.81E-17	4.82E-14
	24 h	mitochondrial protein complex	GO:0098798	1.80E-36	2.32E-33
		mitochondrial part	GO:0044429	1.74E-30	9.10E-28
		membrane-enclosed lumen	GO:0031974	2.11E-30	9.10E-28

		organelle lumen	GO:0043233	3.32E-30	9.54E-28
		intracellular organelle lumen	GO:0070013	3.69E-30	9.54E-28
		mitochondrial inner membrane	GO:0005743	4.08E-29	8.79E-27
		mitochondrial translational termination	GO:0070126	4.31E-29	2.46E-25
		mitochondrial translational elongation	GO:0070125	5.05E-29	2.46E-25
		organelle inner membrane	GO:0019866	5.47E-27	1.01E-24
		mitochondrion	GO:0005739	7.24E-27	1.17E-24
	48 h	sister chromatid segregation	GO:0000819	4.31E-26	3.08E-22
		mitotic cell cycle process	GO:1903047	3.64E-25	1.30E-21
		mitotic sister chromatid segregation	GO:0000070	1.57E-24	3.73E-21
		nuclear chromosome segregation	GO:0098813	2.90E-24	5.18E-21
		mitotic nuclear division	GO:0007067	1.45E-23	2.08E-20
		mitotic cell cycle	GO:0000278	1.87E-23	2.23E-20
		cell cycle	GO:0007049	1.84E-21	1.88E-18
		cell cycle process	GO:0022402	9.48E-20	8.46E-17
		nuclear division	GO:0000280	1.91E-19	1.52E-16
		mitotic cell cycle phase transition	GO:0044772	6.50E-19	4.64E-16
	72 h	mitotic cell cycle process	GO:1903047	6.64E-25	4.30E-21
		mitotic cell cycle	GO:0000278	2.22E-24	7.19E-21
		mitotic cell cycle phase transition	GO:0044772	1.86E-20	4.02E-17
		cell cycle phase transition	GO:0044770	4.18E-19	6.78E-16
		nuclear DNA replication	GO:0033260	9.57E-19	1.11E-15
		sister chromatid segregation	GO:0000819	1.03E-18	1.11E-15
		cell cycle DNA replication	GO:0044786	4.88E-18	4.52E-15
		cell cycle	GO:0007049	6.29E-18	4.80E-15
		nuclear chromosome segregation	GO:0098813	6.66E-18	4.80E-15
		cell cycle process	GO:0022402	1.33E-17	8.63E-15
Liver	0 h	regulation of lipid metabolic process	GO:0019216	0.000082	0.141287
		response to hydrogen peroxide	GO:0042542	0.000099	0.141287
		cellular response to hydrogen peroxide	GO:0070301	0.000146	0.141287
		ovulation from ovarian follicle	GO:0001542	0.000199	0.141287
		steroid metabolic process	GO:0008202	0.000201	0.141287
		response to glucose	GO:0009749	0.000237	0.141287
		response to hexose	GO:0009746	0.000343	0.141287
		pyruvate oxidation	GO:0009444	0.000354	0.141287
		chorionic trophoblast cell differentiation	GO:0060718	0.000354	0.141287
		formation of primary germ layer	GO:0001704	0.000368	0.141287
	6 h	RNA processing	GO:0006396	1.21E-48	1.45E-44
		membrane-enclosed lumen	GO:0031974	3.39E-45	4.52E-42
		intracellular part	GO:0044424	7.18E-45	4.52E-42

	organelle lumen	GO:0043233	8.69E-45	4.52E-42
	intracellular organelle lumen	GO:0070013	1.24E-44	4.86E-42
	intracellular	GO:0005622	2.51E-43	7.84E-41
	rRNA metabolic process	GO:0016072	1.28E-40	7.63E-37
	RNA binding	GO:0003723	3.44E-40	1.06E-36
	rRNA processing	GO:0006364	7.24E-39	2.89E-35
	organic substance metabolic process	GO:0071704	1.38E-38	4.13E-35
12 h	cytoplasmic part	GO:0044444	7.75E-47	1.20E-43
	cytoplasm	GO:0005737	1.67E-45	1.29E-42
	metabolic process	GO:0008152	2.78E-41	3.35E-37
	organic substance metabolic process	GO:0071704	1.50E-39	6.89E-36
	single-organism metabolic process	GO:0044710	1.71E-39	6.89E-36
	small molecule metabolic process	GO:0044281	2.13E-37	5.31E-34
	RNA processing	GO:0006396	2.20E-37	5.31E-34
	membrane-enclosed lumen	GO:0031974	1.74E-36	8.98E-34
	organelle lumen	GO:0043233	4.02E-36	1.55E-33
	ncRNA metabolic process	GO:0034660	9.60E-37	1.93E-33
24 h	cytoplasmic part	GO:0044444	2.78E-43	3.75E-40
	cytoplasm	GO:0005737	3.28E-37	2.22E-34
	single-organism metabolic process	GO:0044710	9.83E-36	1.03E-31
	intracellular membrane-bounded organelle	GO:0043231	3.47E-34	1.56E-31
	membrane-bounded organelle	GO:0043227	2.90E-33	9.82E-31
	mitochondrion	GO:0005739	3.21E-32	8.68E-30
	membrane-enclosed lumen	GO:0031974	4.24E-31	9.55E-29
	organelle lumen	GO:0043233	1.13E-30	2.00E-28
	intracellular organelle lumen	GO:0070013	1.18E-30	2.00E-28
	organonitrogen compound metabolic process	GO:1901564	2.21E-31	1.16E-27
48 h	lipoprotein lipase activity	GO:0004465	1.75E-07	0.00011
	transcription factor activity, sequence-specific DNA binding	GO:0003700	1.86E-07	0.00011
	nucleic acid binding transcription factor activity	GO:0001071	2.21E-07	0.00011
	response to organic cyclic compound	GO:0014070	2.36E-07	0.000936
	negative regulation of transferase activity	GO:0051348	2.80E-07	0.000936
	cellular response to organic cyclic compound	GO:0071407	4.91E-07	0.000955
	negative regulation of transcription, DNA-templated	GO:0045892	6.03E-07	0.000955
	negative regulation of nucleic acid-templated transcription	GO:1903507	7.15E-07	0.000955

	negative regulation of RNA biosynthetic process	GO:1902679	9.56E-07	0.001064
	female pregnancy	GO:0007565	1.22E-06	0.001106
72 h	transcription factor activity, sequence-specific DNA binding	GO:0003700	9.93E-07	0.000636
	nucleic acid binding transcription factor activity	GO:0001071	1.14E-06	0.000636
	MAP kinase phosphatase activity	GO:0033549	1.42E-06	0.000636
	RNA polymerase II regulatory region DNA binding	GO:0001012	8.84E-06	0.001883
	sequence-specific double-stranded DNA binding	GO:1990837	0.00001	0.001883
	sequence-specific DNA binding	GO:0043565	0.000011	0.001883
	double-stranded DNA binding	GO:0003690	0.000011	0.001883
	transcriptional activator activity, RNA polymerase II transcription regulatory region sequence-specific binding	GO:0001228	0.000011	0.001883
	RNA polymerase II regulatory region sequence-specific DNA binding	GO:0000977	0.000016	0.00223
	transcription regulatory region sequence-specific DNA binding	GO:0000976	0.000017	0.00223
