# Supplementary material for: The dynamic immune response of the liver and spleen in leopard coral grouper (Plectropomus leopardus) to Vibrio harveyi infection based on transcriptome analysis
Source: Front Immunol. 2024 Oct 10;15:1457745. doi: 10.3389/fimmu.2024.1457745 (PMC11499110; doi:10.3389/fimmu.2024.1457745)
Supplement: Supplementary file 5 [file DataSheet3.pdf]

Table S3 P value and Q value (Adjusted P value) after KEGG enrichment analysis

Tissue	Up/Down	Time	Pathway	Pathway ID	Pvalue	Qvalue
Spleen	up	0 h	TNF signaling pathway	ko04668	0.000094	0.006053
			Osteoclast differentiation	ko04380	0.000126	0.006053
			Prion disease	ko05020	0.000267	0.008554
		6 h	Ribosome biogenesis in eukaryotes	ko03008	1.18E-19	3.90E-17
			RIG-I-like receptor signaling pathway	ko04622	1.50E-11	2.47E-09
			Cytosolic DNA-sensing pathway	ko04623	4.09E-11	4.50E-09
			Toll-like receptor signaling pathway	ko04620	1.27E-10	1.05E-08
			Legionellosis	ko05134	2.96E-10	1.95E-08
			Influenza A	ko05164	5.55E-10	3.05E-08
			IL-17 signaling pathway	ko04657	3.67E-09	1.72E-07
			Epstein-Barr virus infection	ko05169	4.21E-09	1.72E-07
			Measles	ko05162	4.68E-09	1.72E-07
			TNF signaling pathway	ko04668	5.87E-09	1.94E-07
			Cytokine-cytokine receptor interaction	ko04060	8.51E-09	2.55E-07
			Aminoacyl-tRNA biosynthesis	ko00970	1.39E-08	3.83E-07
			NOD-like receptor signaling pathway	ko04621	2.38E-08	6.05E-07
			Herpes simplex virus 1 infection	ko05168	7.32E-08	1.72E-06
			C-type lectin receptor signaling pathway	ko04625	5.66E-07	0.000012
		12 h	Proteasome	ko03050	6.81E-35	2.20E-32
			Ribosome biogenesis in eukaryotes	ko03008	5.68E-19	9.18E-17
			Aminoacyl-tRNA biosynthesis	ko00970	1.32E-08	1.42E-06
			Spliceosome	ko03040	5.17E-08	4.18E-06
			Antigen processing and presentation	ko04612	2.56E-07	0.000016
			Complement and coagulation cascades	ko04610	2.94E-07	0.000016
			Oxidative phosphorylation	ko00190	2.68E-06	0.000124
			Epstein-Barr virus infection	ko05169	0.00001	0.00042
			Ribosome	ko03010	0.000031	0.001104
			Legionellosis	ko05134	0.000037	0.001198
			RNA degradation	ko03018	0.000073	0.002139
			Nucleocytoplasmic transport	ko03013	0.000086	0.002326
			Collecting duct acid secretion	ko04966	0.000155	0.00384
			Vibrio cholerae infection	ko05110	0.000254	0.005865
			Cysteine and methionine metabolism	ko00270	0.000327	0.007045
		24 h	Oxidative phosphorylation	ko00190	7.66E-39	2.37E-36
			Parkinson disease	ko05012	1.75E-27	2.72E-25
			Proteasome	ko03050	6.12E-25	6.33E-23
			Huntington disease	ko05016	4.15E-19	3.22E-17
			Thermogenesis	ko04714	1.43E-17	8.87E-16
			DNA replication	ko03030	1.34E-13	6.90E-12
			Metabolic pathways	ko01100	1.57E-13	6.94E-12
			Non-alcoholic fatty liver disease	ko04932	1.40E-12	5.42E-11

		Alzheimer disease	ko05010	3.41E-10	1.18E-08
		Ribosome biogenesis in eukaryotes	ko03008	1.74E-08	5.40E-07
		Ribosome	ko03010	1.19E-07	3.35E-06
		Nucleotide excision repair	ko03420	1.31E-06	0.000031
		Spliceosome	ko03040	1.32E-06	0.000031
		Aminoacyl-tRNA biosynthesis	ko00970	2.95E-06	0.000065
		Mismatch repair	ko03430	0.000016	0.000327
	48 h	Cell cycle	ko04110	6.79E-08	0.00001
		Progesterone-mediated oocyte maturation	ko04914	3.77E-06	0.00029
		Glutathione metabolism	ko00480	0.000212	0.010422
		Oocyte meiosis	ko04114	0.000271	0.010422
		Pyrimidine metabolism	ko00240	0.000599	0.018436
	72 h	DNA replication	ko03030	3.64E-21	6.63E-19
		Cell cycle	ko04110	7.41E-21	6.75E-19
		Mismatch repair	ko03430	0.000015	0.000889
		Nucleotide excision repair	ko03420	0.000033	0.001514
		Pyrimidine metabolism	ko00240	0.000161	0.005465
		Progesterone-mediated oocyte maturation	ko04914	0.00018	0.005465
		Cytosolic DNA-sensing pathway	ko04623	0.00026	0.006769
		Base excision repair	ko03410	0.001417	0.029471
		Oocyte meiosis	ko04114	0.001457	0.029471
		Viral protein interaction with cytokine and cytokine receptor	ko04061	0.002279	0.041486
down	0 h	Circadian rhythm - fly	ko04711	7.29E-06	0.001261
		Autophagy - animal	ko04140	0.000097	0.008426
		Chemokine signaling pathway	ko04062	0.000368	0.021206
		Longevity regulating pathway	ko04211	0.000807	0.027196
		FoxO signaling pathway	ko04068	0.000838	0.027196
		Non-alcoholic fatty liver disease	ko04932	0.000943	0.027196
		Notch signaling pathway	ko04330	0.001353	0.033433
		Longevity regulating pathway - multiple species	ko04213	0.001896	0.03644
		Regulation of lipolysis in adipocytes	ko04923	0.001896	0.03644
	6 h	Lysosome	ko04142	2.42E-09	8.09E-07
		Salivary secretion	ko04970	0.000015	0.002545
		Metabolism of xenobiotics by cytochrome P450	ko00980	0.000024	0.002656
		Propanoate metabolism	ko00640	0.000035	0.002902
		Inflammatory mediator regulation of TRP channels	ko04750	0.000143	0.00953
		Metabolic pathways	ko01100	0.000185	0.009543
		Phosphatidylinositol signaling system	ko04070	0.0002	0.009543

	Glucagon signaling pathway	ko04922	0.000282	0.010307
	Other glycan degradation	ko00511	0.000303	0.010307
	Pathways in cancer	ko05200	0.000339	0.010307
	Malaria	ko05144	0.000339	0.010307
	Valine, leucine and isoleucine degradation	ko00280	0.000407	0.011341
	Renin secretion	ko04924	0.000549	0.014094
	Chemical carcinogenesis - DNA adducts	ko05204	0.000801	0.019111
	Parathyroid hormone synthesis, secretion and action	ko04928	0.001086	0.024171
12 h	ECM-receptor interaction	ko04512	2.29E-17	7.42E-15
	PI3K-Akt signaling pathway	ko04151	4.96E-13	8.03E-11
	Focal adhesion	ko04510	2.75E-10	2.97E-08
	Pathways in cancer	ko05200	1.91E-07	0.000015
	Intestinal immune network for IgA production	ko04672	0.000023	0.001504
	Viral protein interaction with cytokine and cytokine receptor	ko04061	0.000038	0.002061
	Amoebiasis	ko05146	0.000064	0.00271
	Malaria	ko05144	0.000067	0.00271
	Small cell lung cancer	ko05222	0.000078	0.002812
	Calcium signaling pathway	ko04020	0.000152	0.004935
	Cell adhesion molecules	ko04514	0.000279	0.00821
	Cytokine-cytokine receptor interaction	ko04060	0.000351	0.009467
	Parathyroid hormone synthesis, secretion and action	ko04928	0.000444	0.011015
	Proteoglycans in cancer	ko05205	0.000532	0.011015
	Salivary secretion	ko04970	0.000538	0.011015
24 h	Intestinal immune network for IgA production	ko04672	4.02E-07	0.000115
	ECM-receptor interaction	ko04512	8.89E-07	0.000127
	PI3K-Akt signaling pathway	ko04151	4.83E-06	0.000461
	Hematopoietic cell lineage	ko04640	0.000031	0.00224
	Viral protein interaction with cytokine and cytokine receptor	ko04061	0.000169	0.009652
	Parathyroid hormone synthesis, secretion and action	ko04928	0.000288	0.013706
	Cytokine-cytokine receptor interaction	ko04060	0.000363	0.014831
	Transcriptional misregulation in cancer	ko05202	0.000562	0.019453
	Focal adhesion	ko04510	0.000612	0.019453
	Cell adhesion molecules	ko04514	0.001296	0.03186
	Pathways in cancer	ko05200	0.001326	0.03186

			Inflammatory mediator regulation of TRP channels	ko04750	0.001337	0.03186
			Th1 and Th2 cell differentiation	ko04658	0.00182	0.04004
		72 h	Circadian rhythm - fly	ko04711	0.000106	0.027498
			Transcriptional misregulation in cancer	ko05202	0.000335	0.04339
			Cholesterol metabolism	ko04979	0.000697	0.048519
			Type II diabetes mellitus	ko04930	0.000754	0.048519
			Viral protein interaction with cytokine and cytokine receptor	ko04061	0.000976	0.048519
			MicroRNAs in cancer	ko05206	0.001301	0.048519
			Nitrogen metabolism	ko00910	0.001311	0.048519
			JAK-STAT signaling pathway	ko04630	0.001508	0.048825
Liver	up	6 h	Spliceosome	ko03040	6.98E-29	2.34E-26
			Proteasome	ko03050	1.65E-24	2.77E-22
			Ribosome biogenesis in eukaryotes	ko03008	3.14E-20	3.51E-18
			Nucleocytoplasmic transport	ko03013	7.53E-17	6.30E-15
			Epstein-Barr virus infection	ko05169	2.89E-12	1.93E-10
			Herpes simplex virus 1 infection	ko05168	2.10E-10	1.17E-08
			Protein export	ko03060	3.45E-09	1.65E-07
			Protein processing in endoplasmic reticulum	ko04141	4.59E-09	1.92E-07
			Toll-like receptor signaling pathway	ko04620	1.67E-08	6.21E-07
			Aminoacyl-tRNA biosynthesis	ko00970	8.25E-08	2.61E-06
			TNF signaling pathway	ko04668	8.58E-08	2.61E-06
			IL-17 signaling pathway	ko04657	1.16E-07	3.23E-06
			Legionellosis	ko05134	3.57E-07	9.20E-06
			RIG-I-like receptor signaling pathway	ko04622	4.21E-07	0.00001
			Cytosolic DNA-sensing pathway	ko04623	5.33E-07	0.000012
		12 h	Spliceosome	ko03040	1.10E-27	2.37E-25
			Ribosome biogenesis in eukaryotes	ko03008	1.43E-27	2.37E-25
			Proteasome	ko03050	1.27E-23	1.41E-21
			Nucleocytoplasmic transport	ko03013	2.31E-15	1.91E-13
			Protein processing in endoplasmic reticulum	ko04141	2.35E-13	1.56E-11
			Aminoacyl-tRNA biosynthesis	ko00970	6.53E-13	3.61E-11
			RNA polymerase	ko03020	5.27E-06	0.00025
			Cytosolic DNA-sensing pathway	ko04623	6.64E-06	0.000276
			mRNA surveillance pathway	ko03015	0.000015	0.000493
			RNA degradation	ko03018	0.000016	0.000493
			Epstein-Barr virus infection	ko05169	0.000016	0.000493
			Legionellosis	ko05134	0.000027	0.000737
			Protein export	ko03060	0.000029	0.000737
			Biosynthesis of amino acids	ko01230	0.000195	0.004627

		ABC transporters	ko02010	0.000646	0.014302
	24 h	Proteasome	ko03050	1.31E-32	4.13E-30
		Protein processing in endoplasmic reticulum	ko04141	5.18E-19	8.19E-17
		Spliceosome	ko03040	2.20E-14	2.32E-12
		Aminoacyl-tRNA biosynthesis	ko00970	4.41E-14	3.48E-12
		Ribosome biogenesis in eukaryotes	ko03008	4.29E-11	2.49E-09
		Protein export	ko03060	4.73E-11	2.49E-09
		Oxidative phosphorylation	ko00190	1.90E-08	8.59E-07
		Nucleocytoplasmic transport	ko03013	4.20E-08	1.66E-06
		Vibrio cholerae infection	ko05110	9.26E-07	0.000032
		RNA degradation	ko03018	1.00E-06	0.000032
		N-Glycan biosynthesis	ko00510	1.75E-06	0.00005
		Biosynthesis of amino acids	ko01230	4.63E-06	0.000122
		RNA polymerase	ko03020	7.02E-06	0.000171
		Various types of N-glycan biosynthesis	ko00513	0.000022	0.000503
		Metabolic pathways	ko01100	0.000092	0.001908
	72 h	Linoleic acid metabolism	ko00591	0.000043	0.006961
		Arachidonic acid metabolism	ko00590	0.000577	0.047064
down	0 h	Steroid biosynthesis	ko00100	0.000058	0.010647
		Viral protein interaction with cytokine and cytokine receptor	ko04061	0.000145	0.013213
	6 h	Metabolic pathways	ko01100	4.22E-08	0.000014
		PPAR signaling pathway	ko03320	1.11E-06	0.000182
		Glucagon signaling pathway	ko04922	4.72E-06	0.000513
		Fatty acid degradation	ko00071	7.70E-06	0.000627
		Parathyroid hormone synthesis, secretion and action	ko04928	0.000023	0.001528
		Starch and sucrose metabolism	ko00500	0.000072	0.003887
		Circadian rhythm - fly	ko04711	0.00012	0.0056
		Insulin resistance	ko04931	0.000138	0.005634
		AMPK signaling pathway	ko04152	0.000156	0.005664
		Insulin signaling pathway	ko04910	0.000198	0.006436
		Bile secretion	ko04976	0.000236	0.006436
		Carbon metabolism	ko01200	0.000237	0.006436
		Glycine, serine and threonine metabolism	ko00260	0.000301	0.007554
		Glycolysis / Gluconeogenesis	ko00010	0.000361	0.008403
		One carbon pool by folate	ko00670	0.000434	0.009423
	12 h	Metabolic pathways	ko01100	1.30E-28	4.35E-26
		ECM-receptor interaction	ko04512	1.90E-12	3.18E-10
		Carbon metabolism	ko01200	6.21E-12	6.91E-10
		Protein digestion and absorption	ko04974	1.83E-11	1.53E-09
		Glycolysis / Gluconeogenesis	ko00010	1.11E-10	7.44E-09

	Focal adhesion	ko04510	1.37E-10	7.65E-09
	Drug metabolism - cytochrome P450	ko00982	2.48E-10	1.18E-08
	Metabolism of xenobiotics by cytochrome P450	ko00980	1.00E-09	4.19E-08
	Pancreatic secretion	ko04972	1.05E-08	3.92E-07
	Tryptophan metabolism	ko00380	2.11E-08	7.03E-07
	Linoleic acid metabolism	ko00591	2.74E-08	8.31E-07
	Chemical carcinogenesis - DNA adducts	ko05204	5.12E-08	1.43E-06
	Glyoxylate and dicarboxylate metabolism	ko00630	6.64E-08	1.71E-06
	Tyrosine metabolism	ko00350	1.50E-07	3.57E-06
	Primary bile acid biosynthesis	ko00120	2.30E-07	5.13E-06
24 h	Protein digestion and absorption	ko04974	8.84E-15	2.86E-12
	Pancreatic secretion	ko04972	2.07E-13	3.36E-11
	Complement and coagulation cascades	ko04610	9.37E-11	1.01E-08
	Drug metabolism - cytochrome P450	ko00982	1.35E-10	1.10E-08
	Metabolic pathways	ko01100	4.77E-09	3.09E-07
	Metabolism of xenobiotics by cytochrome P450	ko00980	2.00E-07	0.000011
	Linoleic acid metabolism	ko00591	2.99E-07	0.000014
	Chemical carcinogenesis - DNA adducts	ko05204	5.25E-07	0.000021
	ECM-receptor interaction	ko04512	6.57E-06	0.000237
	Tyrosine metabolism	ko00350	0.000019	0.000607
	Fat digestion and absorption	ko04975	0.000062	0.001831
	Primary bile acid biosynthesis	ko00120	0.000092	0.002483
	Glycolysis / Gluconeogenesis	ko00010	0.00013	0.003243
	Focal adhesion	ko04510	0.000267	0.006171
	Purine metabolism	ko00230	0.000362	0.00748
48 h	Pancreatic secretion	ko04972	3.63E-14	1.03E-11
	Protein digestion and absorption	ko04974	5.52E-07	0.000078
	Glycerolipid metabolism	ko00561	0.000034	0.003203
	Fat digestion and absorption	ko04975	0.000065	0.00448
	Insulin resistance	ko04931	0.000079	0.00448
	Cellular senescence	ko04218	0.000372	0.015355
	Circadian rhythm - fly	ko04711	0.000378	0.015355
	Steroid biosynthesis	ko00100	0.000657	0.023334
	Chronic myeloid leukemia	ko05220	0.000756	0.023848
	Glucagon signaling pathway	ko04922	0.001536	0.040097
	Linoleic acid metabolism	ko00591	0.001641	0.040097
	MAPK signaling pathway	ko04010	0.001694	0.040097
	Pathways in cancer	ko05200	0.002121	0.043906
	PI3K-Akt signaling pathway	ko04151	0.002164	0.043906

	Transcriptional misregulation in cancer	ko05202	0.002367	0.044809
72 h	Pancreatic secretion	ko04972	5.61E-13	1.55E-10
	Colorectal cancer	ko05210	3.13E-06	0.000432
	Endometrial cancer	ko05213	0.000071	0.005919
	Protein digestion and absorption	ko04974	0.00009	0.005919
	Fat digestion and absorption	ko04975	0.000107	0.005919
	MAPK signaling pathway	ko04010	0.000157	0.007199
	Apoptosis	ko04210	0.000398	0.015682
	FoxO signaling pathway	ko04068	0.000541	0.01768
	Apoptosis - fly	ko04214	0.0006	0.01768
	ErbB signaling pathway	ko04012	0.000703	0.01768
	Cellular senescence	ko04218	0.000711	0.01768
	Glycerolipid metabolism	ko00561	0.000769	0.01768
	IL-17 signaling pathway	ko04657	0.000994	0.021094
	Thyroid cancer	ko05216	0.001372	0.027031
	p53 signaling pathway	ko04115	0.001469	0.027031
	Non-small cell lung cancer	ko05223	0.001757	0.030303
